# Scavenger receptor BI promotes cytoplasmic accumulation of lipoproteins in clear-cell renal cell carcinoma[S]

**DOI:** 10.1194/jlr.M083311

**Published:** 2018-09-01

**Authors:** Srividya Velagapudi, Peter Schraml, Mustafa Yalcinkaya, Hella A. Bolck, Lucia Rohrer, Holger Moch, Arnold von Eckardstein

**Affiliations:** Institute of Clinical Chemistry* University of Zurich and University Hospital of Zurich, Zurich, Switzerland; Department of Pathology and Molecular Pathology,† University of Zurich and University Hospital of Zurich, Zurich, Switzerland

**Keywords:** low density lipoprotein, high density lipoprotein, vascular endothelial growth factor

## Abstract

Clear-cell renal cell carcinomas (ccRCCs) are characterized by inactivation of the von Hippel-Lindau (VHL) gene and intracellular lipid accumulation by unknown pathomechanisms. The immunochemical analysis of 356 RCCs revealed high abundance of apoA-I and apoB, as well as scavenger receptor BI (SR-BI) in the ccRCC subtype. Given the characteristic loss of VHL function in ccRCC, we used VHL-defective and VHL-proficient cells to study the potential influence of VHL on lipoprotein uptake. VHL-defective patient-derived ccRCC cells and cell lines (786O and RCC4) showed enhanced uptake as well as less resecretion and degradation of radio-iodinated HDL and LDL (^125^I-HDL and ^125^I-LDL, respectively) compared with the VHL-proficient cells. The ccRCC cells showed enhanced vascular endothelial growth factor (VEGF) and SR-BI expression compared with normal kidney epithelial cells. Uptake of ^125^I-HDL and ^125^I-LDL by patient-derived normal kidney epithelial cells as well as the VHL-reexpressing ccRCC cell lines, 786-O-VHL and RCC4-O-VHL cells, was strongly enhanced by VEGF treatment. The knockdown of the VEGF coreceptor, neuropilin-1 (NRP1), as well as blocking of SR-BI significantly reduced the uptake of lipoproteins into ccRCC cells in vitro. LDL stimulated proliferation of 786-O cells more potently than 786-O-VHL cells in a NRP1- and SR-BI-dependent manner. In conclusion, enhanced lipoprotein uptake due to increased activities of VEGF/NRP1 and SR-BI promotes lipid accumulation and proliferation of VHL-defective ccRCC cells.

Classification of renal cell carcinoma (RCC) subtypes is based on histologically predominant cytoplasmic features [clear-cell RCC (ccRCC)], characteristic staining (chromophobe RCC), architectural features (papillary RCC), or specific molecular alterations (translocation RCC). ccRCC received its name from the microscopic appearance upon staining of formalin-fixed paraffin-embedded (FFPE) sections with H&E (1). The clear appearance of the cytoplasm is due to the accumulation of glycogen and lipids that are dissolved during routine processing with deparaffinization of FFPE sections using xylene and ethanol. The most prominent lipid stored in renal tumor cells is cholesterol, largely in the esterified form (2). The mechanisms for cholesterol accumulation in ccRCC cells are not well-understood. Three principle pathways have to be considered, two of which have been ruled out previously, namely, excessive cholesterol synthesis by the finding of decreased rather than increased activity of the rate-limiting enzyme, HMG-CoA reductase (3), as well as abnormal cholesterol efflux (2). The third explanation is the most likely, excessive uptake of cholesterol from plasma lipoproteins beyond the capacity of utilization and processing. However, neither the lipoprotein classes nor the receptors and cellular pathways involved are well-characterized. ccRCC lacks the LDL receptor (LDLR), which is the main entry route for exogenous cholesterol into the majority of cells, including many tumor cells (4). In contrast, the expression of both the VLDL receptor (VLDLR) and scavenger receptor BI (SR-BI) was found to be increased in ccRCC compared with the normal kidney tissue (5), and to mediate lipid uptake into ccRCC cells from VLDL and HDL, respectively (5, 6).

The activity of vascular endothelial growth factor (VEGF) is increased in the majority of ccRCCs (7, 8) due to the constitutive activation of hypoxia-inducible factor (HIF)-1α by somatic mutations in the von Hippel-Lindau (*VHL*) tumor suppressor gene. The VHL protein is a component of the E3-ubiquitin ligase complex that ubiquitylates HIF-1α and HIF-2α for proteasome-mediated degradation (7–9). Thus, the loss of *VHL* function leads to HIF-1α stabilization despite an adequately oxygenated tissue microenvironment, which in turn results in uncontrolled activation of HIF-target genes that regulate erythropoiesis (erythropoietin), angiogenesis (VEGF), glycolysis (glucose transporters and glycolytic pathway enzymes), and apoptosis (BNIP3) (8–12). We have previously found that VEGF promotes the cell surface abundance of SR-BI in endothelial cells and thereby enhances the uptake of HDL into endothelial cells (13). Therefore, we hypothesized that increased activities of HIF-1α and hence VEGF promote the cell surface expression of SR-BI and thereby the uptake of HDL. To test this hypothesis, we combined immunohistochemical studies in human renal tumors with experiments in two ccRCC model cell lines and patient-derived ccRCC cell cultures.

## MATERIALS AND METHODS

### Patients, tissue microarray construction, and immunohistochemistry

RCC patients were identified from the database of the Institute of Pathology and Molecular Pathology, University Hospital Zurich, Switzerland. All RCCs were histologically reevaluated by one pathologist (H.M.) and selected on the basis of H&E-stained tissue sections. The patient cohort and the construction of tissue microarrays (TMAs) of RCC were previously described (14, 15). Tumors were staged and histologically classified according to the World Health Organization classification (16). Overall survival data were obtained by the Cancer Registry of the Canton Zurich. The clinical and pathologic parameters of the tumors on the TMA are summarized in supplemental Table S1. For some cases, there was no information available. This study was approved by the local commission of ethics (KEK-ZH no. 2011-0072/4).

TMA sections (2.5 μm) were transferred to glass slides followed by immunohistochemical analysis according to the Ventana (Tucson, AZ) automated protocols, and the antibodies used are listed in supplemental Table S2. The staining intensities were classified as absent (0), weak (1), moderate (2), and strong (3). For detailed analysis, TMAs were scanned using the NanoZoomer digital slide scanner (Hamamatsu Photonics K.K.).

### Cell culture

Tissue samples of patients were made available by the Tissue Biobank of the Department of Pathology and Molecular Pathology, University Hospital of Zurich, Switzerland upon approval of the local ethics commission (KEK-ZH-Nr. 2011-0072 and KEK-ZH-Nr. 2014-0614) and upon patients’ written consent. H&E-stained sections of FFPE and fresh-frozen renal tissue specimens were reviewed by a pathologist with specialization in uropathology (H.M.). Sanger sequencing was employed to assess the mutation status of the *VHL* gene (c.341-1G>C) for the ccRCC primary tumor and the corresponding cell culture. DNA was isolated from FFPE punches from tumor tissue (three cylinders with a diameter of 0.6 mm) or a minimum of 10,000 cultured cells using the Maxwell® 16 DNA purification kits (Promega, Madison, WI). PCR and sequencing of *VHL* were performed as previously described (17). Fresh tissue samples were placed into sterile 50 ml conical tubes containing transport medium (RPMI) (Gibco, Waltham, MA) with 10% FCS (Gibco) and Antibiotic-Antimycotic® (Gibco). FFPE cell pellets from cultured cells were prepared as previously described (18) and compared with FFPE specimens of the corresponding primary tumor by immunohistochemistry. Cultures were maintained in K1 medium (19, 20) supplemented with 0.5% FCS (Gibco) and epinephrine (Sigma-Aldrich, St. Louis, MO) and transferred into collagen I-coated cell culture dishes (Corning, NY) in a humidified incubator at 37°C with 5% CO_2_.

The ccRCC-derived 786-O cells, which lack functional pVHL, were supplied by ATCC and cultured in RPMI-1640 (Sigma; R8758) with 10% fetal bovine serum (Gibco), 100 U/ml of penicillin, and 100 μg/ml of streptomycin (Sigma-Aldrich). Stable transfectant of 786-O reexpressing pVHL-isoform 30 (786-O-VHL) was provided by Prof. Dr. Wilhelm Krek (ETH Zurich), generated as described (21) and cultured using the same conditions as mentioned for 786-O. G418 (0.5 mg/ml) (Gibco; 10131) was used as selection antibiotic. Both cell lines were authenticated by the authentication service of Microsynth (Balgach, Switzerland) and were previously used by our group (22, 23).

Human aortic endothelial cells (HAECs) from Cell Applications Inc. (304-05a) were cultured in endothelial cell basal medium (LONZA Clonetics CC-3156) with 5% fetal bovine serum (Gibco), 100 U/ml of penicillin, and 100 μg/ml of streptomycin (Sigma-Aldrich), supplemented with singleQuots (LONZA Clonetics CC-4176 or ATCC PCS-100-041). Hepatocellular carcinoma cells (Huh7) from JCRB (0403) and human renal proximal tubular epithelial cell line, HK-2 (provided by R. Wüthrich Clinic for Nephrology, Department of Internal Medicine, University Hospital Zurich, Switzerland), were cultured in DMEM with 10% fetal bovine serum (Gibco), 100 U/ml of penicillin, and 100 μg/ml of streptomycin (Sigma-Aldrich).

### Lipoprotein isolation and labeling

LDL (1.019 < d < 1.063 g/ml) and HDL (1.063 < d < 1.21 g/ml) were isolated from fresh normolipidemic plasma of blood donors by sequential ultracentrifugation as described previously (24, 25). LDL and HDL were radioiodinated with Na^125^I by the McFarlane monochloride procedure modified for lipoproteins (25, 26). Specific activities between 300–900 cpm/ng of protein were obtained.

### Lipoprotein cell association, pulse-chase, and degradation assays

All assays were performed in RPMI-1640 (Sigma) containing 25 mmol/l HEPES and 0.2% BSA instead of serum (referred to as assay medium). Where indicated, cells were pretreated with sorafenib tosylate (Selleckchem; 90 nM) or sunitinib malate (Selleckchem; 80 nM) for 30 min or with VEGF-A (Sigma; 25 ng/ml) or anti-SR-BI neutralizing antibody (1:500; Novus NB400-113) or anti-IgG control (1:500, Santa Cruz-2027) for 1 h at 37°C. Following treatments, the cells were incubated with 10 μg/ml of radio-iodinated HDL (^125^I-HDL) or radio-iodinated LDL (^125^I-LDL) in the absence or presence of a 40 times excess of nonlabeled HDL and LDL, respectively, for 1 h at 37°C for association experiments. At the end of the cell association step, the cells were washed twice with Tris-BSA buffer, followed by two washes with PBS containing CaCl_2_ and MgCl_2_ and then lysed in 0.1 N NaOH buffer. Specific cellular association was calculated by subtracting the values obtained in the presence of excess unlabeled HDL or LDL (unspecific) from those obtained in the absence of unlabeled HDL and LDL (total), respectively.

For the pulse-chase experiments, 50,000 cells were seeded in 24-well plates and cultured for 48 h. Then the cells were pulsed for 1 h with 10 μg/ml of ^125^I-HDL or ^125^I-LDL at 37°C in the presence or absence of the respective unlabeled lipoprotein for competition and determination of specific interactions. After 1 h of pulse incubation, the cells were either directly processed for the measurement of association or were washed three times with assay medium, chased for 1, 2, or 4 h at 37°C with the assay medium containing 10 μg/ml of unlabeled HDL or LDL. At the end of each chase period, the cells were handled as described above for the cellular association experiments. In addition, the media were collected and subjected to precipitation with trichloroacetic acid (TCA). Radioactivity was counted by Perkin Elmer γ-counter. Precipitated radioactivity was postulated to reflect nondegraded lipoproteins, whereas radioactivity in the supernatant was considered to reflect degraded lipoproteins (27). The amount of radioactivity in each fraction of the well (cell associated, TCA supernatant, and TCA precipitated) was calculated by normalizing to the specific cellular association of the no chase of parental 786-O cells or primary ccRCC cells (represents initial radioactivity for the chase points).

### Real-time PCR

Total RNA was isolated using TRI reagent (Sigma T9424) according to the manufacturer’s instructions. Genomic DNA was removed by digestion using DNase (Roche) and RNase inhibitor (Ribolock; Thermo Scientific). Reverse transcription was performed using M-MLVRT (Invitrogen; 200 U/μl) following the standard protocol as described by the manufacturer. Quantitative PCR was done with Lightcycler FastStart DNA Master SYBR Green I (Roche) using gene-specific primers, as mentioned in the supplemental information.

### siRNA transfection

The 786-O and 786-O-VHL cells were reverse transfected with siRNA (Ambion Silencer Select; Life Technologies) targeted to LDLR (s224006, s224007, s4), VLDLR [siGENOME SMARTpool siRNA D-003721-02; ON-TARGET plus human VLDLR (7436), Dharmacon], neuropilin-1 (NRP1) (s16844, s16843), or nonsilencing control (4390843, silencer select or siGENOME control siRNA D-001220-01-20, Dharmacon or ON-TARGET control siRNA D-01810-10-20, Dharmacon) at a final concentration of 5 nmol/l using Lipofectamine RNA iMAX transfection reagent (Invitrogen; 13778150) in an antibiotic-free medium. All experiments were performed 72 h posttransfection and efficiency of transfection was confirmed with at least two siRNAs against each gene using quantitative RT-PCR.

### Western blotting

Cells were lysed in RIPA buffer [10 mmol/l Tris (pH 7.4), 150 mmol/l NaCl, 1% NP-40, 1% sodium deoxycholate, 0.1% SDS, and complete EDTA (Roche)] with protease inhibitors (Roche). Equal amounts of protein were separated on SDS-PAGE and trans-blotted onto PVDF membrane (GE Healthcare). Membranes were blocked in appropriate blocking buffer recommended for the antibody (TBS-T supplemented with 5% milk) and incubated overnight on a shaker at 4°C with primary antibodies in the same blocking buffer. Membranes were incubated for 1 h with a HRP-conjugated secondary antibody (Dako) in the blocking buffer. Membranes were further incubated with chemiluminescence substrate for 1 min (Pierce ECL Plus; Thermo Scientific) and imaged using Fusion Fx (Vilber). The expression of LDLR (1:1,000, ab30532; Abcam), VLDLR (1:1,000, NBP1-78162; Novus), SR-BI (1:1,000, NB400-131; Novus), and NRP1 (1:1,000, ab81321; Abcam) were evaluated and compared with the expression of TATA binding protein (TBP) (1:1,000, ab51841; Abcam), which was used as a loading control.

### Cell surface expression analysis

Biotinylation of intact cells was performed using 20 mg/ml EZ-Link Sulfo-NHS-S-S-Biotin (Thermo Scientific) in the cold for 1 h with mild shaking and quenched with ice-cold 50 mM Tris (pH 7.4). Cells were lysed in RIPA buffer (total cell lysate), and 200–500 μg of lysates were incubated with 20 μl of BSA-blocked streptavidin beads suspension (GE Healthcare) for 16 h at 4°C and pelleted by centrifugation; the pellet represents surface proteins. Proteins were dissociated from the pellet by boiling with SDS loading buffer, analyzed by SDS-PAGE, and immunoblotted with SR-BI antibody (NB400-131; Novus), TBP (ab51841; Abcam) used as intracellular control, and Na^+^/K^+^-ATPase (1:200, Santa Cruz-21712) used as cell surface control.

### Cell proliferation assay

Cells were cultured at a density of 5,000 cells per well in a 96-well plate for 72 h. After transfecting with either siRNA against NRP1 or LDLR or nonsilencing controls for 60 h or blocking with SR-BI neutralizing antibody for 1 h, the cells were treated overnight with 50 μg/ml of HDL or LDL. Following the overnight treatment, the supernatant was removed and cells were washed twice with PBS. The cells were then incubated with 30 μl of MTT solution (5 mg/ml in PBS, M5655; Sigma) diluted in 270 μl of DMEM for 30 min. The resultant formazan salts were extracted with DMSO and absorbance intensity was read at 550 nm and reference wavelength at 650 nm (DMSO). The rate of cell proliferation was calculated relative to the 786-O parental cell line.

### Statistical analysis

Contingency table analysis and Pearson’s chi-square tests were used to analyze the associations between protein expression patterns and clinical parameters. Overall survival rates were determined according to the Kaplan-Meier method and analyzed for statistical differences using a log-rank test.

The data sets for all in vitro experiments were performed with the GraphPad Prism 7.02 software. Data sets from independent experiments were pooled and the statistical tests were chosen based on the number of groups being compared (two or more than two). All the in vitro tests in this work are based either on the Mann-Whitney *t*-test or Kruskal-Wallis followed by Dunn’s posttest. Values are expressed as mean ± SEM. *P* < 0.05 was regarded as significant and *P* > 0.05 was regarded as not significant.

## RESULTS

### Lipoprotein and apolipoprotein expression and pathological parameters

**Fig. 1.**
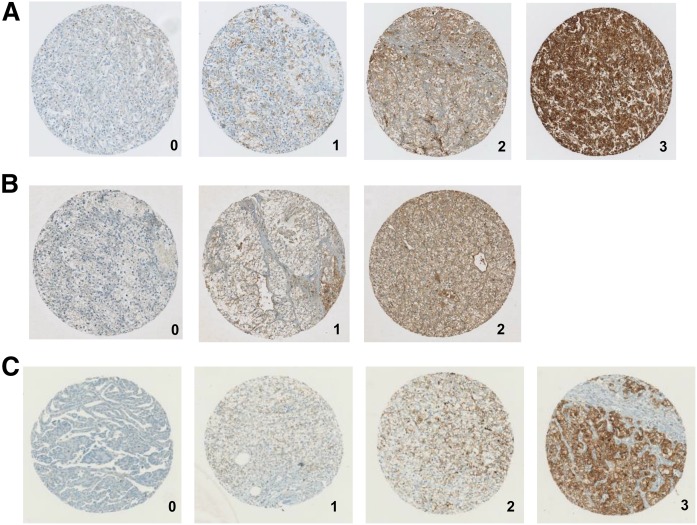
Apolipoprotein and SR-BI expression in RCC TMA immunostaining of apoA-I (A), apoB (B), and SR-BI (C) of human TMAs. The grading from 0 to 3 represents the staining intensity from negative to the strongest.

Immunostaining was performed on RCC TMAs for the major apolipoproteins of HDL and LDL, apoA-I and apoB, respectively (**Fig. 1A**, B), as well as SR-BI (Fig. 1C). Based on the staining intensities, expression levels in tumors were graded from 0 to 3 for apoA-I and SR-BI expression, and from 0 to 2 for apoB expression, as staining intensities were generally lower. Increased immunoreactivity with anti-apoA-I or anti-SR-BI antibodies, but not immunoreactivity with anti-apoB antibodies, significantly differentiated 175 ccRCC tissues from papillary RCC (**Table 1**).

**TABLE 1.** Quantification of apolipoproteins and SR-BI expression in RCC TMA

Protein of Interest	Staining Intensity	Subtype		
		ccRCC [% (n)]	Chromophobe [% (n)]	Papillary [% (n)]
apoA-I	0	5.1 (9)	0	12 (3)
	1	18.9 (33)	50 (3)	44 (11)
	2	24.6 (43)	16.7 (1)	32 (8)
	3	51.4 (90)	33.3 (2)	12 (3)
apoB	0	21.7 (38)	16.7 (1)	24 (6)
	1	62.3 (109)	50 (3)	68 (17)
	2	16 (28)	33.3 (2)	8 (2)
SR-BI	0	18.7 (31)	80 (4)	78.9 (15)
	1	25.3 (42)	20 (1)	10.5 (2)
	2	27.7 (46)	0	5.3 (1)
	3	28.3 (47)	0	5.3 (1)

Percent and absolute (n) frequencies of anti-apoA-I, anti-apoB, and anti-SR-BI staining intensities among RCC subtypes.

Strong cytoplasmic apoA-I and SR-BI expression (staining intensity of 2 and 3) was seen in approximately 75% and 56% of ccRCCs. Moderate and weak cytoplasmic apoB expression was observed in 78% of ccRCCs. These data indicate that the presence of apolipoproteins is characteristic in the majority of ccRCCs (Table 1).

We next evaluated the associations of the lipoprotein immunoreactivities in ccRCCs with tumor stage (pT) and ISUP grade. Only anti-apoB immunoreactivity was significantly associated with late tumor stage (supplemental Table S3, *P* = 0.0371). apoA-I (*P* = 0.025) and apoB (*P* = 0.0006), but not SR-BI, expression was significantly correlated with higher ISUP grade (supplemental Table S4). We used the Kaplan-Meier method and log-rank test to evaluate any associations of apoA-I, apoB, and SR-BI immunoreactivity with overall survival (supplemental Fig. S1a–c). Only anti-apoA-I expression was significantly correlated with worse patient outcome (*P* = 0.0407, supplemental Fig. S1a). This association lost statistical significance upon multivariate analysis taking into account tumor stage and grade.

### Expression of apolipoproteins, SR-BI, and VHL downstream targets

Loss of function of the VHL protein leads to stabilization of HIF-α in ccRCC. Therefore, we statistically evaluated the associations of lipoprotein immunoreactivity with markers of the VHL/HIF axis, namely HIF-1α (supplemental Table S5) and HIF-1α targets CA9 and GLUT1 (supplemental Tables S6, S7), as well as microvessel density recorded by CD34 abundance (supplemental Table S8). Interestingly, the immunoreactivity for apoA-I showed significant positive associations with each of the four markers (HIF-1α, *P* = 0.0078; CA9, *P* = 0.0272; GLUT1, *P* = 0.0175; and CD34, *P* < 0.001). apoB immunoreactivity was significantly and positively associated with microvessel density (*P* = 0.0197) and nuclear HIF-1α staining (*P* = 0.0049). However, SR-BI immunoreactivity showed no significant association with any marker.

### ccRCC cells do not express apoA-I and apoB, but show enhanced uptake and impaired degradation and resecretion of both HDL and LDL

To unravel the origin of lipoprotein accumulation in ccRCC, we performed in vitro assays in two ccRCC cell lines that lack functional VHL (786-O and RCC4) and their stably transfected VHL wild-type counterparts (786-O-VHL, RCC4-O-VHL). To further corroborate our findings, we utilized patient-derived ccRCC and normal epithelial kidney cell cultures that were established from surgical tissue specimens. Histological and genotypic comparison verified the resemblance of the patient-derived ccRCC and normal epithelial cell cultures to their respective primary human tissue. Immunohistochemistry of Pax8 and pan-cytokeratin verified the renal epithelial origin of both PDC cultures, while CAIX expression unequivocally confirmed the presence of malignant cells in the ccRCC PDC culture (supplemental Fig. S2). Importantly, the ccRCC-derived cell culture retained the *VHL* driver mutation of the primary human tumor.

We first analyzed by RT-PCR to determine whether *APOA1* and *APOB* are expressed by patient-derived ccRCC cells or the ccRCC cell lines. The hepatocellular-derived carcinoma cell line, Huh7, was used as positive control; HAECs served as negative control (supplemental Fig. S3a, b). Neither patient-derived ccRCC cells nor 786-O nor RCC4 showed any APOA1 and APOB transcripts (supplemental Fig. S3). However, we found both anti-apoA-I and anti-apoB immunoreactivity in ccRCC tissue and patient-derived cell culture, possibly due to uptake from the serum-containing cell culture media.

**Fig. 2.**
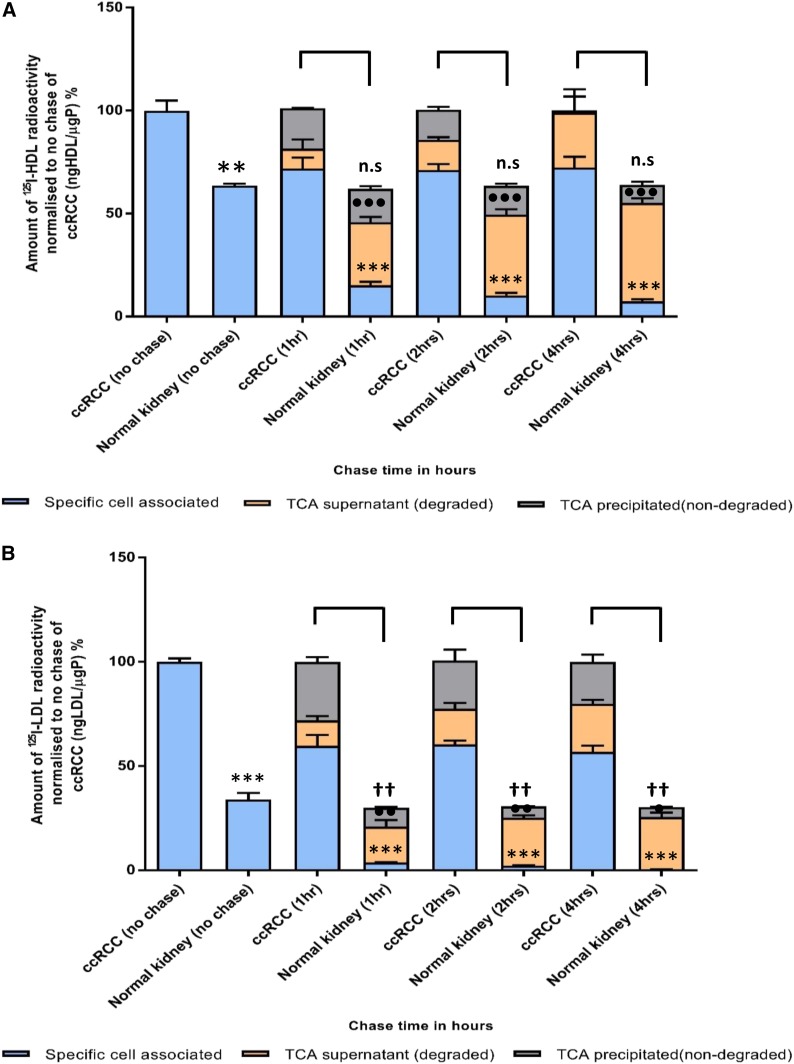
Cellular association, resecretion, and degradation of ^125^I-HDL and ^125^I-LDL in patient-derived ccRCC and normal kidney epithelial cell cultures. Both ccRCC- and normal kidney-derived cells were pulsed for 1 h with 10 μg/ml of either ^125^I-HDL (A) or ^125^I-LDL (B) at 37°C in the absence (total) or in the presence of a 40-fold excess of unlabeled HDL or LDL (unspecific). Subsequently, the cells were either lysed and analyzed for their content of radiolabel or chased for 1, 2, and 4 h with unlabeled HDL or LDL at 37°C. The media were collected and subjected to precipitation with TCA to count the radioactivity of nondegraded /resecreted lipoproteins in the precipitate and degraded lipoproteins in the supernatant separately. Specific association was calculated by subtracting unspecific values from total values. The amount of radioactivity in each fraction of the well (cell associated, TCA supernatant, and TCA precipitated) was calculated by normalizing to the specific cellular association of the no chase of ccRCC cells (represents initial radioactivity for the chase points). The results are represented as the mean ± SEM of two independent experiments with two batches of ^125^I-HDL and ^125^I-LDL. Significance was determined for each fraction at each time point using the Mann-Whitney test. For specific cell associated: ***P ≤ 0.001; for TCA supernatant: •••*P* ≤ 0.001, ••*P* ≤ 0.01, •*P* ≤ 0.05; for TCA precipitated: ††*P* ≤ 0.01, †*P* ≤ 0.05. n.s, not significant.

**Fig. 3.**
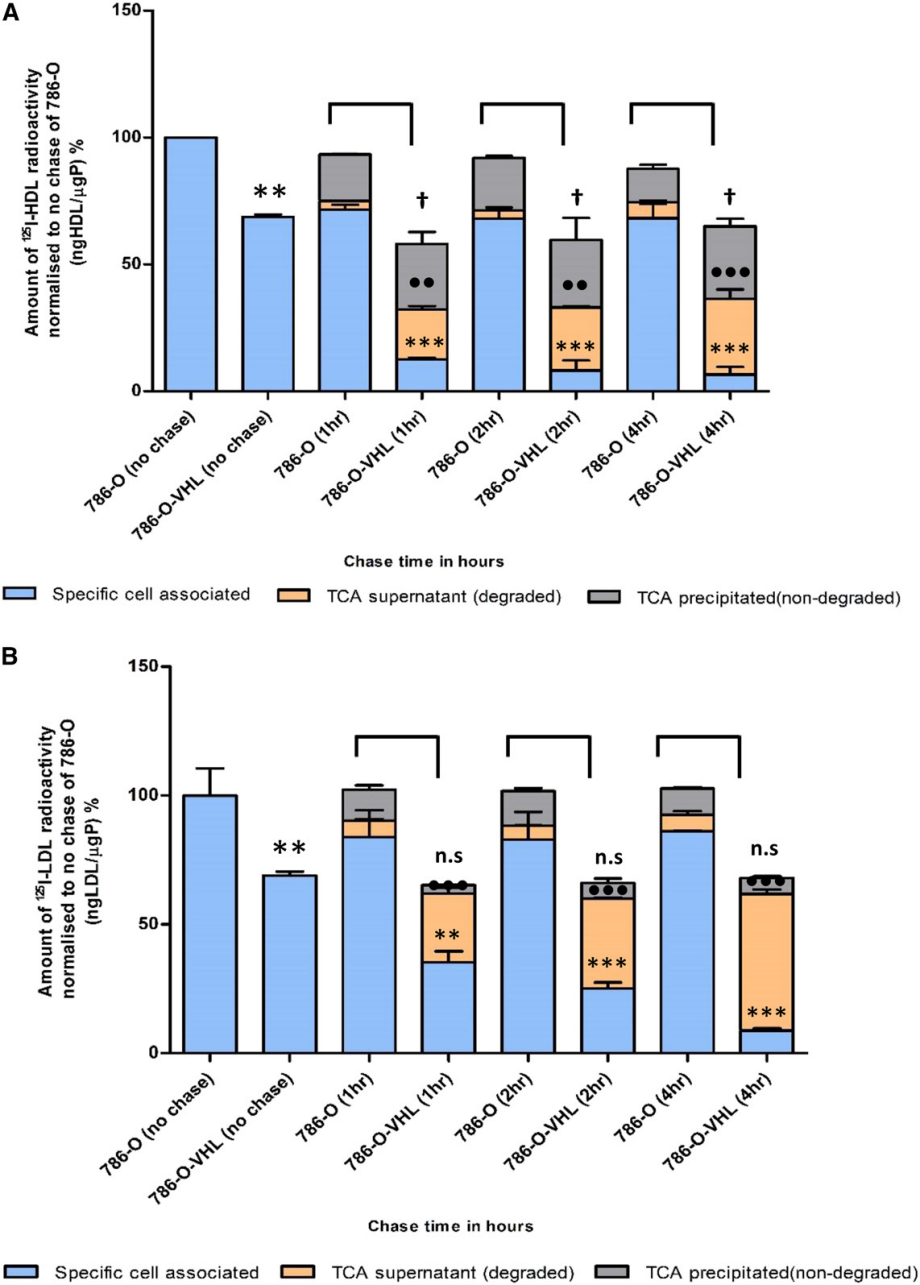
Cellular association, resecretion, and degradation of ^125^I-HDL and ^125^I-LDL in 786-O and 786-O-VHL cells. The results are represented as the mean ± SEM of two independent experiments, with two batches of ^125^I-HDL (A) and ^125^I-LDL (B), which were performed as described in the legend of Fig. 2. Significance was determined for each fraction at each time point using the Mann-Whitney test. For specific cell associated: ***P ≤ 0.001, **P ≤ 0.01; for TCA supernatant: •••*P* ≤ 0.001, ••*P* ≤ 0.01; for TCA precipitated: †*P* ≤ 0.05. n.s, not significant.

Both the patient-derived ccRCC cell culture and the VHL-deficient cell lines, 786-O and RCC4, showed significantly higher specific cell association of ^125^I-HDL and ^125^I-LDL compared with the normal kidney epithelial cells and the retransfected counterpart cell lines, 786-O-VHL and RCC4-VHL: After 1 h of incubation and compared with cultured normal epithelial kidney cells, association of ^125^I-HDL and ^125^I-LDL were 50–100% and 80–150% higher, respectively, in the ccRCC-derived cell culture (first two columns of **Fig. 2A**, B as well as supplemental Fig. S4a, b). The differences in cell association of ^125^I-HDL and ^125^I-LDL between 786-O and 786-O-VHL amounted to 40–200% and 70–100%, respectively (first two columns of **Fig. 3A**, B as well as supplemental Fig. S4c, d). The association of ^125^I-HDL and ^125^I-LDL by RCC4 and RCC4-VHL even differed by factors 5 and 10, respectively (supplemental Fig. S4e, f). This indicates that loss of pVHL correlates with increased lipoprotein uptake by ccRCC cells.

To investigate whether the increased cellular association of ^125^I-HDL and ^125^I-LDL by VHL-deficient cells is caused by impaired degradation or resecretion of the cell-associated lipoproteins, a combination of pulse-chase and degradation experiments were performed with ^125^I-HDL and ^125^I-LDL. Already after 1 h chase, only 24% and 9% of the initially cell-associated ^125^I-HDL and ^125^I-LDL, respectively, remained associated with cultured normal kidney epithelial cells. After 4 h, both decreased significantly over prolonged chasing time to about 10% and 1% of the initial radioactivity, respectively. During prolonged chase, the percentage of degraded ^125^I-HDL increased to 70% at the expense of the nondegraded, which decreased to 15% (Fig. 2A, supplemental Fig. S5a). Very strikingly, in the patient-derived ccRCC cell culture, the percentage of cell-associated ^125^I-HDL was as high as 70% after 1 h chase and did not decrease significantly over prolonged chase. The percentages of degraded ^125^I-HDL increased over time so that after 4 h chase no radioactivity was precipitated in the medium. At any time point of chase, the radioactivity of both degraded and nondegraded ^125^I-HDL was significantly lower in the media of patient-derived ccRCC cells compared with the media of normal kidney cells (Fig. 2A, supplemental Fig. S5a).

The radioactivity of ^125^I-LDL in the medium of cultured normal kidney cells showed similar kinetics to that of ^125^I-HDL, except that the proportion of non-TCA-precipitable radioactivity was already higher after 1 h chase (60%) and increased to 80% after 4 h chase. The proportion of the nondegraded ^125^I-LDL decreased from 30% after 1 h chase to 15% after 4 h chase. Compared with patient-derived normal kidney cells, the ccRCC cell culture was significantly less active in degrading ^125^I-LDL: the non-TCA-precipitable proportion of radioactivity was only less than 15% after 1 h chase and increased to 25% after 4 h chase. The relative difference in TCA-precipitable radioactivity between patient-derived normal kidney and ccRCC cells was significant, but less prominent (Fig. 2B, supplemental Fig. S5b).

In principle, the same observations were made for the kinetics of ^125^I-HDL and ^125^I-LDL in a pulse chase experiment in the ccRCC cell lines, 786-O and 786-O-VHL (Fig. 3, supplemental Fig. S6): the 786-O-VHL cells showed considerable degradation of ^125^I-HDL and ^125^I-LDL as well as resecretion of ^125^I-HDL, whereas the parental 786-O failed to degrade both ^125^I-HDL and ^125^I-LDL. The 786-O cells also showed a strongly reduced capacity to resecrete ^125^I-HDL (Fig. 3A, B; supplemental Fig. S6a, b). Together, these results indicate that the loss of VHL function in ccRCC cells increases lipoprotein uptake into a nondegrading nonsecretory compartment.

### VEGF and NRP1 promote the cellular association of ^125^I-HDL and ^125^I-LDL in ccRCC

**Fig. 4.**
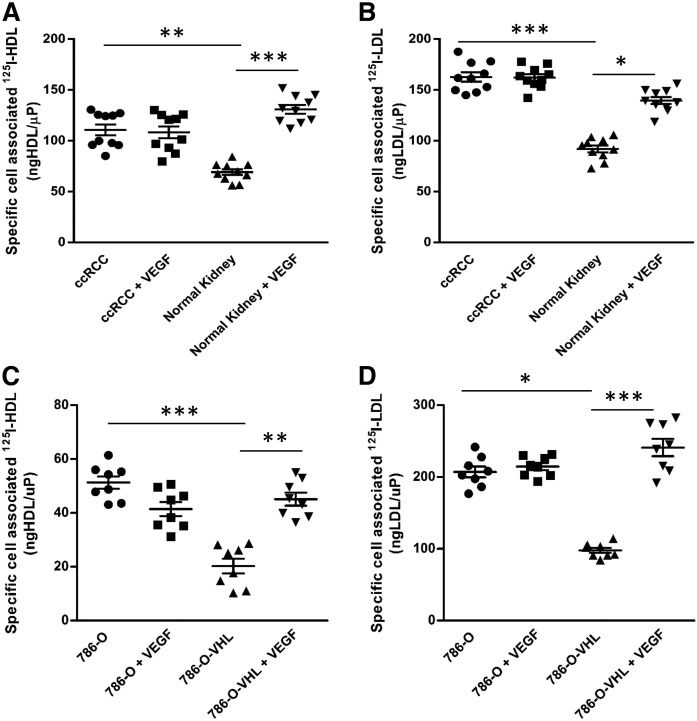
Loss of VHL promotes cellular association of ^125^I-HDL and ^125^I-LDL in ccRCC cells. Patient-derived ccRCC (ccRCC) and normal epithelial kidney (Normal Kidney) cell cultures (786-O and 786-O-VHL cells) were pretreated with 25 ng/ml of VEGF (+ VEGF) for 1 h prior to assays, followed by incubation with 10 μg/ml of ^125^I-HDL (A, C) or ^125^I-LDL (B, D) at 37°C for 1 h in the absence (total) or in the presence of a 40-fold excess of unlabeled HDL or LDL (unspecific). Specific cellular association was calculated by subtracting unspecific values from total values. The results are represented as the mean ± SEM of three independent experiments (in triplicate, triplicate, and duplicate, respectively) for cell lines and two independent experiments with patient-derived cells, with two batches of ^125^I-HDL and ^125^I-LDL. Significance was determined by Kruskal-Wallis test followed by Dunn’s posttest. ***P ≤ 0.001, **P ≤ 0.01, *P ≤ 0.05.

As expected, the patient-derived ccRCC cell culture as well as 786-O and RCC4 cells showed higher VEGF mRNA expression compared with patient-derived normal kidney epithelial cells, 786-O-VHL and RCC4-O-VHL cells, respectively (supplemental Fig. S7). Pretreatment with VEGF for 1 h increased the cellular association of both ^125^I-HDL and ^125^I-LDL in patient-derived normal epithelial kidney cells, but not in ccRCC-derived cells (**Fig. 4A**, B). Similar observations were made in the ccRCC cell lines: The significantly lower cellular association of both ^125^I-HDL and ^125^I-LDL by 786-O-VHL and RCC4-O-VHL cells was increased by pretreatment with VEGF for 1 h to the same level as observed in 786-O (Fig. 4C, D) and RCC4 cells (supplemental Fig. S8a, b), respectively, with or without treatment with VEGF. Further, supporting the crucial role of VEGF, pretreatment of 786-O cells with VEGF receptor (VEGFR) inhibitors for 30 min decreased specific cellular association of both ^125^I-HDL and ^125^I-LDL, but had no effect in 786-O-VHL cells (supplemental Fig. S9a–d).

**Fig. 5.**
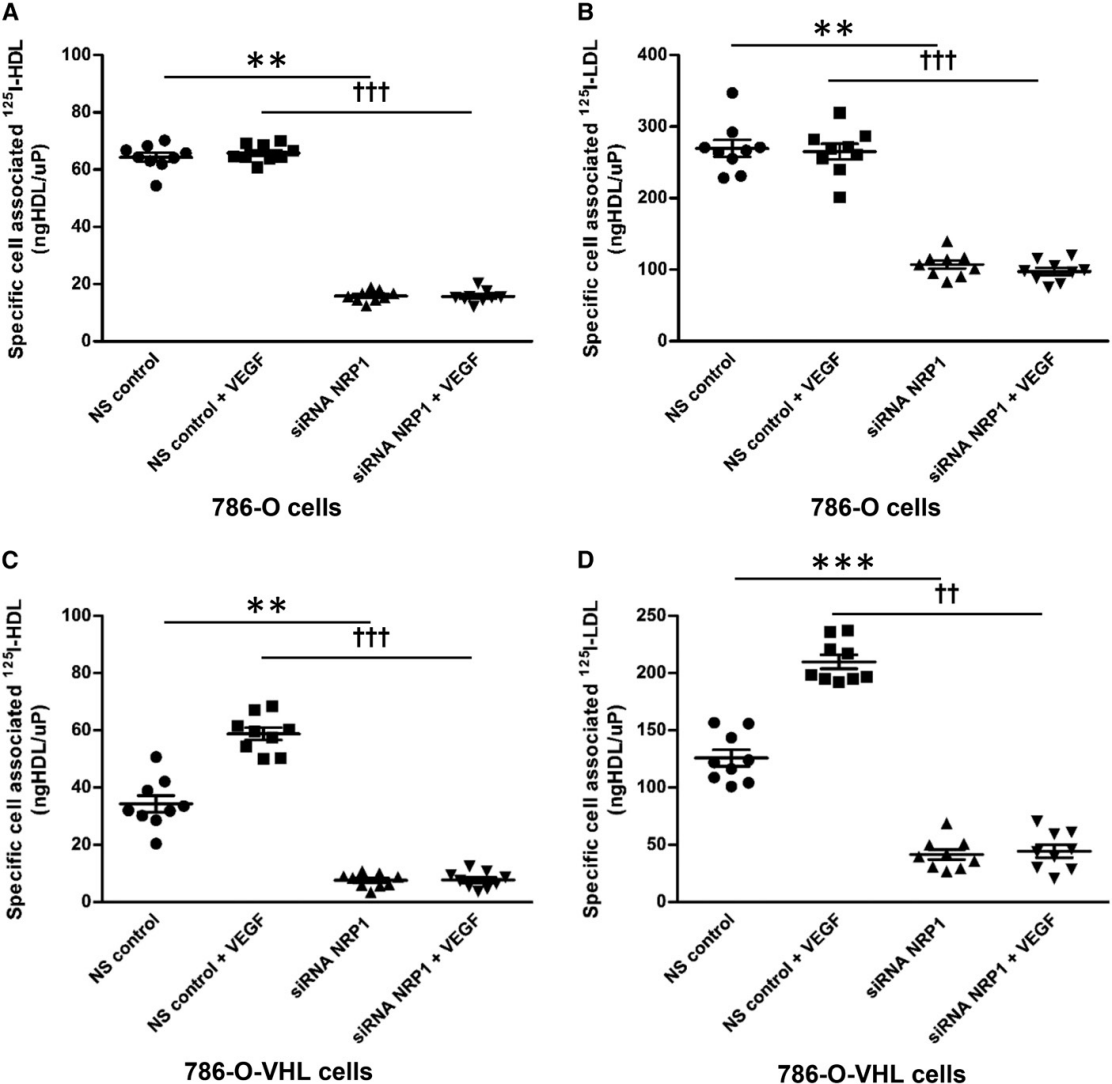
NRP1 promotes cellular association of ^125^I-HDL and ^125^I-LDL in renal carcinoma 786-O cells. The 786-O and 786-O-VHL cells were transfected with specific siRNA against NRP1 or with nonsilencing control siRNA (NS control). After 72 h of transfection, the cells were pretreated with 25 ng/ml of VEGF for 1 h prior to assays (as indicated), followed by incubation with 10 μg/ml of ^125^I-HDL or ^125^I-LDL. The 786-O cells were incubated with ^125^I-HDL (A) and ^125^I-LDL (B), and the 786-O-VHL cells were incubated with ^125^I-HDL (C) and ^125^I-LDL (D) at 37°C for 1 h in the absence (total) or in the presence of a 40-fold excess of unlabeled HDL or LDL (unspecific). Specific cellular association was calculated by subtracting unspecific values from total values. The results are represented as the mean ± SEM of three independent experiments (each experiment was performed in triplicate) with two batches of ^125^I-HDL and ^125^I-LDL. Significance was determined by Kruskal-Wallis test followed by Dunn’s posttest. ***P ≤ 0.001, **P ≤ 0.01, †††*P* ≤ 0.001, ††*P* ≤ 0.01.

Interestingly, ccRCC cells did not express VEGFR1 (*FLT1*), VEGFR2 (*KDR*), or VEGFR3 (*FLT4*), but expressed *NRP1*, which is a coreceptor of VEGFR (supplemental Table S9). To determine whether NRP1 mediates the effects of VEGF on the cellular association of radioiodinated lipoproteins in vitro, we targeted NRP1 using RNAi in 786-O and 786-O-VHL cells (supplemental Fig. S10a, b). Silencing of NRP1 led to decreased specific cellular association of ^125^I-HDL as well as ^125^I-LDL in both 786-O and 786-O-VHL cells. Interestingly, pretreatment with VEGF for 1 h did not stimulate the specific cellular association of either ^125^I-HDL or ^125^I-LDL in 786-O-VHL cells in which NRP1 was knocked down (**Fig. 5A**–D). In conclusion, VEGF/NRP1-mediated signaling stimulates the uptake of ^125^I-HDL as well as ^125^I-LDL in ccRCC.

### SR-BI, but not LDLR or VLDLR, mediates cellular association of ^125^I-HDL and ^125^I-LDL in ccRCC

**Fig. 6.**
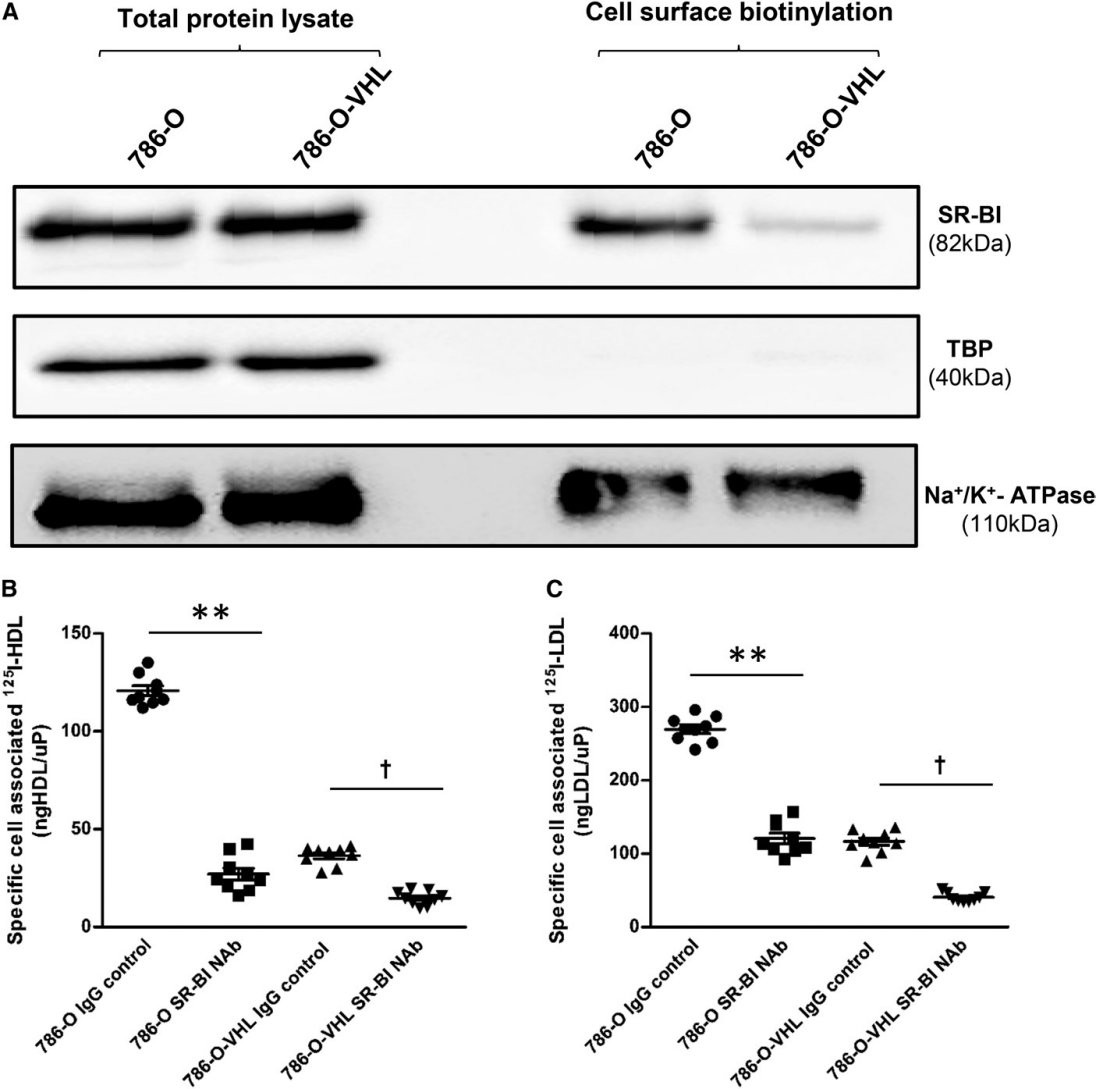
SR-BI mediates cellular association of ^125^I-HDL and ^125^I-LDL in 786-O cells. A: Cell surface expression of SR-BI in renal carcinoma cells. Western blot analysis of SR-BI in total cell lysates (left) and on the cell surface (right) in 786-O and 786-O-VHL cells. The Western blots were probed with anti-SR-BI (82 kDa), anti-TBP (40 kDa, used as a control for intracellular protein expression), and anti-Na^+^/K^+^-ATPase (110 kDa, used as a control for cell surface protein expression). The 786-O and 786-O-VHL cells were pretreated with either anti-SR-BI neutralizing antibody or isotype (IgG) control for 1 h prior to assays, followed by incubation with 10 μg/ml of ^125^I-HDL (B) or ^125^I-LDL (C) for 1 h in the absence (total) or in the presence of 40-fold excess of unlabeled HDL or LDL (unspecific) at 37°C. Specific association was calculated by subtracting unspecific values from total values. The results are represented as the mean ± SEM of three independent experiments (each experiment performed in triplicate) with two batches of ^125^I-HDL and ^125^I-LDL. Significance was determined by Kruskal-Wallis test followed by Dunn’s posttest. IgG control represents isotype control and anti-SR-BI Nab represents treatment with SR-BI neutralizing antibody. **P ≤ 0.01, †*P* ≤ 0.05. Complete blots are in supplemental Electrophoretic Blot SA.

**Fig. 7.**
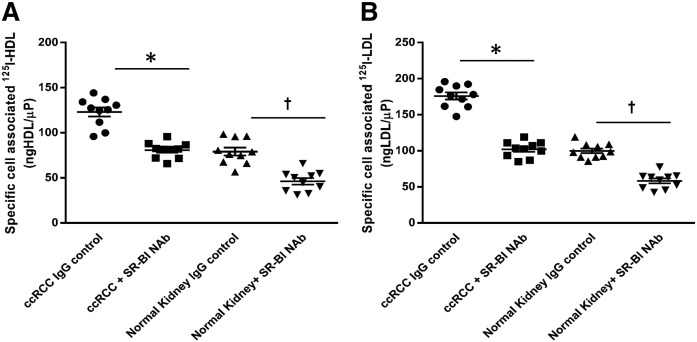
SR-BI mediates cellular association of ^125^I-HDL and ^125^I-LDL in ccRCC cells. Both the patient-derived ccRCC and normal epithelial kidney cell cultures were pretreated with either anti-SR-BI neutralizing antibody (Nab) or isotype control (IgG control) for 1 h prior to assays, followed by incubation with 10 μg/ml of ^125^I-HDL (A) or ^125^I-LDL (B) for 1 h in the absence (total) or in the presence of a 40-fold excess of unlabeled HDL or LDL (unspecific) at 37°C. Specific association was calculated by subtracting unspecific values from total values. The results are represented as the mean ± SEM of two independent experiments (each experiment performed in triplicate) with two batches of ^125^I-HDL and ^125^I-LDL. Significance was determined by Kruskal-Wallis test followed by Dunn’s posttest. IgG control represents isotype control and anti-SR-BI Nab represents treatment with SR-BI neutralizing antibody. *P ≤ 0.05, †*P* ≤ 0.05.

We next aimed to unravel which receptor enhances the lipoprotein uptake by ccRCC cells. We first assessed the expression of the candidate receptors, LDLR, VLDLR, and SR-BI, in vitro. We found lower expression of LDLR in the 786-O cells compared with the 786-O-VHL cells (supplemental Fig. S11a). By contrast, total lysates of 786-O and 786-O-VHL cells did not differ in SR-BI or VLDLR protein levels (**Fig. 6A**, supplemental Fig. S12a). However, cell-surface biotinylation revealed strongly increased expression of SR-BI on the cell surface of parental 786-O cells compared with that of 786-O-VHL cells (Fig. 6A). Knockdowns of LDLR and VLDLR by RNAi efficiently suppressed protein abundance of LDLR and VLDLR, respectively (supplemental Figs. S11b, S12a), but did not affect the cellular association of iodinated lipoproteins (supplemental Figs. S11c, S12b, c). By contrast, in both 786-O and 786-O-VHL cells, the specific cellular association of ^125^I-HDL as well as ^125^I-LDL was significantly decreased in the presence of a neutralizing anti-SR-BI-antibody (Fig. 6B, C). The neutralizing anti-SR-BI-antibody also decreased the cellular association of ^125^I-HDL and ^125^I-LDL by RCC4 and RCC4-O-VHL cells (supplemental Fig. S13a, b), as well as in patient-derived ccRCC and normal kidney epithelial cell cultures (**Fig. 7A**, B). Taken together, these results indicate that the uptake of HDL and LDL by ccRCC cells depends on the cell-surface expression of SR-BI.

### NRP1 and SR-BI mediate LDL-dependent proliferation of ccRCC cells

**Fig. 8.**
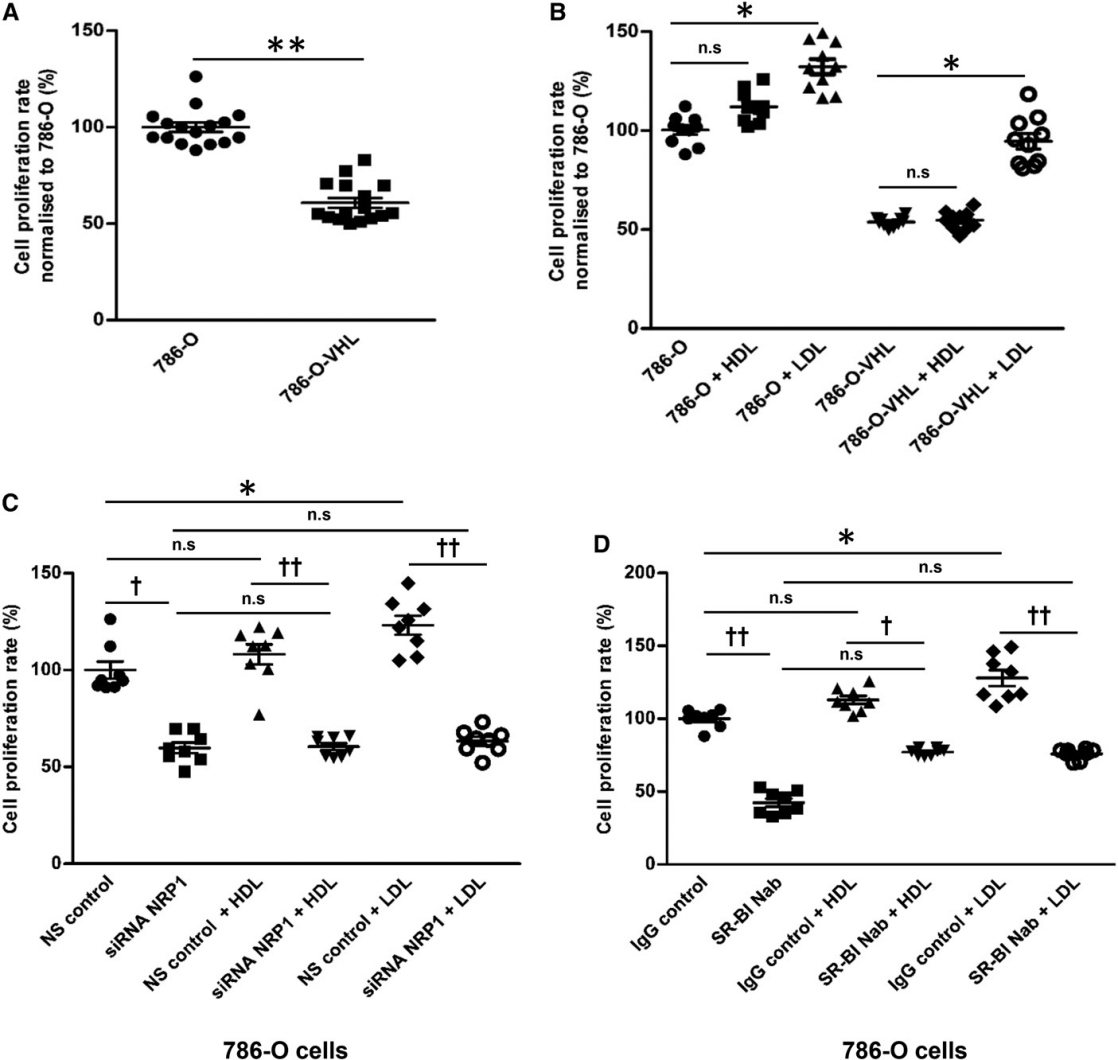
Effects of VHL, HDL, LDL, NRP1, and SR-BI on proliferation of ccRCC cells. VHL-defective 786-O cells and VHL-intact 786-O-VHL cells were cultured in a 96-well plate for 60 h prior to the overnight treatment with 50 μg/ml of HDL or LDL. The rate of proliferation was analyzed using the MTT assay. The comparison of 786-O and 786-O-VHL cells per se (A) or in the presence or absence of 50 μg/ml of either HDL or LDL (B) is shown. The 786-O cells were transfected with specific siRNA against NRP1 or with nonsilencing control siRNA (NS control) for 60 h (C) or pretreated with a neutralizing antiSR-BI antibody for 1 h prior to the overnight treatment with 50 μg/ml of HDL or LDL (D). The results are represented as the mean ± SEM of three independent experiments with two batches of HDL and LDL. Significance was determined by Kruskal-Wallis test followed by Dunn’s posttest. IgG control represents isotype control and anti-SR-BI Nab represents treatment with neutralizing anti-SR-BI antibody. ***P ≤ 0.001, **P ≤ 0.01, *P ≤ 0.05; †††*P* ≤ 0.001, ††*P* ≤ 0.01, †*P* ≤ 0.05. n.s, not significant.

We finally investigated whether the excess cellular association and accumulation of lipoproteins contributed to the proliferation of ccRCC cells. We found higher proliferation of 786-O cells compared with 786-O-VHL cells, indicating that loss of VHL function increases proliferation in ccRCC cells (**Fig. 8A**). Interestingly, pretreatment with LDL, but less so with HDL, increased the proliferation of both ccRCC cell lines (Fig. 8B). Basal as well as lipoprotein-stimulated proliferation was reduced in both 786-O and 786-O-VHL cells by knockdown of NRP1 (Fig. 8C, supplemental Fig. S14a). Both in the presence and absence of lipoproteins and in both 786-O and 786-O-VHL cells (supplemental Fig. S14b–d), the proliferation was significantly decreased by the neutralization of SR-BI (Fig. 8D, supplemental Fig. S14b), but not by knockdown of LDLR (supplemental Fig. S14c, d). Together, these results indicate that LDL promotes the proliferation of ccRCC cells in a NRP1- and SR-BI-dependent manner, but not in a LDLR-dependent manner.

## DISCUSSION

By combining immunohistochemical studies of renal carcinomas with functional experiments in three ccRCC cell culture models, we made several important findings on the as yet little understood mechanism and pathogenic role of intracellular lipid accumulation in ccRCC. First, the immunochemical investigation of apoA-I and apoB in renal carcinomas revealed that ccRCCs store lipoproteins rather than lipids per se. Second, our functional comparisons of a patient-derived ccRCC with a normal epithelial kidney cell culture as well as two VHL-defective ccRCC cell lines (786-O and RCC4) with VHL-intact derivative cell lines (786-O-VHL and RCC4-VHL, respectively) identified enhanced uptake of LDL and HDL and subsequent impairment of degradation and resecretion as the likely mechanism leading to intracellular lipid accumulation in ccRCC. Third, SR-BI was identified as the rate-limiting lipoprotein receptor for the association of both HDL and LDL by ccRCC cells. Fourth VEGF, which is highly expressed by ccRCCs, was found to promote the association of lipoproteins by ccRCC cells through activation of its noncanonical receptor, NRP1. Fifth, LDL was found to promote ccRCC cell proliferation in a SR-BI- and NRP1-dependent manner.

The intracellular storage of LDL is very unusual because the internalization of LDL into other cells, notably hepatocytes and macrophages, but also renal mesangial or tubular cells, is followed by degradation (27–29). Most cells internalize LDL via the LDLR into clathrin-coated pits (30, 31). LDL is then trafficked into an endosomal/lysosomal route, depending on the presence or absence of PCSK9 either together with or separated from the receptor (32, 33). The apoB moiety and cholesteryl esters of LDL are hydrolyzed by lysosomal proteases and acid lipase, respectively (34–36). In hepatocytes and macrophages, and also in other cells, any cholesteryl ester storage in lipid droplets results from the reesterification of cholesterol in the ER after transfer from the lysosomes (37). The intracellular storage of endocytosed holoparticles hence argues against any role of the LDLR for lipid storage in ccRCC. In fact, we confirmed the finding of others that LDLR is suppressed in ccRCC and ruled out (by RNAi) that LDLR contributes to LDL or HDL association by ccRCC cells (supplemental Fig. S11). Internalization of apoB-containing lipoproteins by other members of the LDLR family, for example LRP1, LRP2, or VLDLR, into a broad variety of cells is also followed by lysosomal degradation of both their proteins and lipids (38). Interestingly, the VLDLR was previously reported to be upregulated in ccRCC and to promote lipid uptake into ccRCC cells. However, this study only recorded the uptake of VLDL-derived lipids rather than the lipoproteins’ protein moiety (5). We found no difference in the expression of the VLDLR between the ccRCC cell lin, 786-O, and its VHL-expressing counterpart, 786-O-VHL. Moreover, our siRNA experiments ruled out that the VLDLR contributes to the uptake of LDL or HDL by ccRCC (supplemental Fig. S12).

The most likely reason for the unusual lipoprotein storage by ccRCC cells is the involvement of SR-BI. We, like others (5, 6, 39), found strong immunoreactivity of SR-BI in ccRCC, but not in other renal tumors. Interestingly, Xu et al. (6) previously reported a reduced content of HDL-cholesterol in 786-O cells that were treated with siRNA against SR-BI. We here extend these previous findings by showing that the inhibition of SR-BI prevents the uptake of LDL as well as HDL into patient-derived cultured ccRCC cells as well as into the 786-O and RCC4 cell lines (Figs. 6B, C; 7A, B). The mechanism by which SR-BI promotes cellular lipoprotein uptake is not clear: SR-BI is traditionally regarded as a receptor that binds both HDL and LDL and provides bidirectional fluxes of cholesterol from these lipoproteins into cells or from the plasma membrane to the lipoprotein depending on the concentration gradient (40, 41). However, several examples have been reported where ablation or blockage of SR-BI also inhibited the uptake of lipoprotein particles. Notably, vascular endothelial cells were reported by our laboratory and other laboratories to internalize and transcytose HDL and LDL in a SR-BI-dependent manner (42, 43). However, it is not clear whether SR-BI or one of its splice variants directly serves as an endocytic receptor (44, 45) or whether SR-BI only enables other pathways of endocytosis. Such indirect effects of SR-BI may include the activation of other receptors by altering the cholesterol distribution within the plasma membrane or signaling via its PDZ domain (46). SR-BI-mediated endocytosis of HDL and LDL into endothelial cells is followed by resecretion and, hence, allows transcytosis of lipoproteins, for example from the blood stream into the arterial wall or into the brain as well as from the extravascular tissue into the lymph (13, 47, 48). Interestingly, we here found that a patient-derived ccRCC cell culture as well as the VHL-deficient 786-O and RCC4 cell lines not only fail to degrade but also resecrete the internalized lipoproteins, notably HDL.

VEGF signaling, activated by loss of VHL function, appears to be the reason for the enhanced SR-BI-mediated uptake of HDL and LDL into ccRCC cells. Compared with VHL-proficient normal kidney cells, 786-O-VHL cells, and RCC4-VHL cells, the VHL-deficient ccRCC-derived 786-O and RCC4 cells show increased expression of VEGF (supplemental Fig. S7a–c). Pretreatment with VEGF increased the uptake of both HDL and LDL by normal kidney cells as well as VHL retransfected cells, which are characterized by low endogenous VEGF expression. However, VEGF did not alter the uptake of lipoproteins by the patient-derived ccRCC cell culture nor 786-O or RCC4 cells, which already express high amounts of VEGF (Fig. 4A–D, supplemental Fig. S8a, b). Conversely, lipoprotein uptake was lowered in 786-O cells by VEGFR inhibitors (supplemental Fig. S9a–d). These findings are in line with the previous report of HIF-1α-dependent lipid uptake into ccRCC (6). They are also in line with our previous observation in HAECs where VEGF promoted the translocation of SR-BI to the cell membrane as well as the uptake and transcytosis of HDL (13). The significant correlation of VHL/HIF-1α downstream targets (Glut1 and CAIX) with immunoreactivity of both apoA-I and apoB also suggests that increased cellular apoA-I and apoB levels are a consequence of VHL/HIF-1α signaling activation. Activated VEGF signaling in ccRCC due to increased HIF-1α activity may enhance lipoprotein uptake into the tumor not only by direct actions on tumor cells but also by indirectly promoting their transport from the circulation into the tumor tissue. VEGF is known to activate the downstream signaling by binding to VEGFRs (49). However, we did not detect the expression of any of the three VEGFRs in the ccRCC cells (supplemental Table S9). Importantly, in line with previous findings (50), we detected higher expression of NRP1 in the VHL-lacking 786-O cells compared with the wild-type VHL-expressing 786-O-VHL cells. Upon binding of VEGF, NRP1 elicits angiogenesis and tumorigenesis both dependently and independently of VEGFRs. The enhanced uptake of HDL and LDL in 786-O-VHL cells by pretreatment with VEGF was abrogated by the suppression of NRP1 (Fig. 5). Altogether, our findings identify a novel role of NRP1 in the cholesterol accumulation of ccRCC.

Our observations provide a plausible explanation for the origin of lipid accumulation in ccRCC. However, they do not allow any conclusion to be drawn on whether enhanced lipoprotein uptake into ccRCC has any impact on the clinical course of this disease. Excessive lipids in cancer cells are considered to be markers of cancer aggressiveness (51). In line with this, immunoreactivity for apoA-I, apoB, or SR-BI was associated with the differentiation of renal carcinomas into ccRCC as well as with tumor grade (apoA-I and apoB) or tumor stage (apoB). However, expression of apoB or SR-BI showed no association with prognosis. By contrast, apoB and apoA-I immunoreactivities were associated with lower tumor stage and better survival, respectively (supplemental Table S3, supplemental Fig. S1). Previously, in two Chinese studies each encompassing about 100 patients, SR-BI protein and mRNA expression were found to be associated with the prognosis of ccRCC (6, 52). We did not replicate this observation in our larger cohort of 172 patients. However, it is important to note that, in our in vitro experiments, the cell surface expression of SR-BI, rather than the total SR-BI content, was dependent on VHL and VEGF. The semi-quantitative scoring of immunostaining intensity does not, however, discriminate between the larger pool of intracellular SR-BI and the smaller pool of cell surface SR-BI. Interestingly, genome-wide association studies identified a borderline significant association of the rs4765623 polymorphism in SCARB1 with ccRCC susceptibility (53), indicating a pathogenic role of SR-BI in ccRCC. In line with an oncogenic role, knockdown of SR-BI was found to inhibit proliferation (Fig. 9 in Ref. 6), colony formation, migration, and invasion of ccRCC cells as well as expression and phosphorylation of Akt (6). SR-BI has also been associated with carcinogenic features in breast cancer, prostate cancer, and melanoma (54, 55).

In conclusion, we identified SR-BI-mediated intracellular accumulation of intact lipoproteins as the likely origin of cholesterol accumulation and the characteristic clear cytoplasm of ccRCC. VEGF-induced SR-BI cell surface translocation may be the underlying mechanism and the resulting enhanced SR-BI/lipoprotein interaction may contribute to proliferation and, hence, the prognosis of ccRCC.

## Supplementary Material

Supplemental Data
