# Supplementary material for: Scavenger receptor BI promotes cytoplasmic accumulation of lipoproteins in clear-cell renal cell carcinoma
Source: J Lipid Res. 2018 Sep 1;59(11):2188–201. doi: 10.1194/jlr.M083311 (PMC6210910; doi:10.1194/jlr.M083311)
Supplement: Supplemental Data [file supp_59_11_2188__index.html]

Scavenger receptor BI promotes cytoplasmic accumulation of lipoproteins in clear-cell renal cell carcinoma — Scavenger receptor BI promotes cytoplasmic accumulation of lipoproteins in clear-cell renal cell carcinoma — Supplemental Data 

# Scavenger receptor BI promotes cytoplasmic accumulation of lipoproteins in clear-cell renal cell carcinoma

## Supplemental Data

- Supplementary material (.pdf, 2.6 MB) - This file contains all the supplementary tables and figures corresponding to the main manuscript.
