# Supplementary material for: Scavenger receptor BI promotes cytoplasmic accumulation of lipoproteins in clear-cell renal cell carcinoma
Source: J Lipid Res. 2018 Sep 1;59(11):2188–201. doi: 10.1194/jlr.M083311 (PMC6210910; doi:10.1194/jlr.M083311)
Supplement: Supplemental Data [file 10.1194_M083311_jlr.M083311-1.pdf]

Scavenger receptor BI promotes cytoplasmic accumulation of lipoproteins in clear-cell renal cell carcinoma

Srividya Velagapudi[1], Peter Schraml[2], Mustafa Yalcinkaya[1], Hella A. Bolck[2], Lucia Rohrer[1], Holger Moch[2,*], and Arnold von Eckardstein[1,*]

***: equal contribution**

Institute of Clinical Chemistry[1] and Department of Pathology and Molecular Pathology[2], University of Zurich and University Hospital of Zurich, Switzerland

Supplementary Material

SUPPLEMENTAL MATERIAL AND METHODS

Real-time polymerase chain reaction

Following gene specific primers were used for the real-time polymerase chain reaction:

APOA1 (For: ATGAAAGCTGCGGTGCTG; Rev: AGGTCCTTCACTCGATCCCA)

APOB (For: TGCCTCTCCTGGGTGTTCTA; Rev: CCCGAAGGCTGAAATGGTCT)

VEGF (For: CTGTCTAATGCCCTGGAGCC; Rev: ACGCGAGTCTGTGTTTTTGC)

LDL-R (For: AAGGACACAGCACACAACCA; Rev: CATTTCCTCTGCCAGCAACG)

SCARB1 (For: CTGTGGGTGAGATCATGTGG; Rev: GCCAGAAGTCAACCTTGCTC)

BNIP3 (For: GGAAGATGATATTGAAAGAAGGAAAG, Rev: CGCCTTCCAATATAGATCCCC)

SLC2A1 (For: ACTGTCGTGTCGCTGTTTG, Rev: CCAGGACCCACTTCAAAGAA)

PDK1 (For: CACGCTGGGTAATGAGGATT, Rev: GGAGGTCTCAACACGAGGT)

CAIX (For: GGGTGTCATCTGGACTGTGTT, Rev: CTTCTGTGCTGCCTTCTCATC)

VEGFR1 (For: CTGAAGGAAGGGAGCTCGTC, Rev: GGCGTGGTGTGCTTATTTGG)

VEGFR2 (For: CGGTCAACAAAGTCGGGAGA, Rev: CAGTGCACCACAAAGACACG)

VEGFR3 (For: TCCTACGTGTTCGTGAGAGAC, Rev: CACCAGGAAGGGGTTGGAAA)

NRP1 (For: AGGACAGAGACTGCAAGTATGAC, Rev: AACATTCAGGACCTCTCTTGA)

GAPDH (For: CCCATGTTCGTCATGGGTGT; Rev: TGGTCATGAGTCCTTCCACGA TA).

Supplementary Table S1

Clinical parameters	Patients (%)
**RCC subtype**	
Clear-cell	264
Papillary	48
Chromophobe	15
Oncocytoma	19
Other	8
**pT stage***	
1	102
2	26
3	128
4	7
**ISUP grading***	
1	5
2	68
3	89
4	101
**Sex***	
Women	96
Men	168
**Events***	
Censor	133
Death	107

Supplementary Table S1: Clinicopathological characteristics of patients and tumors The clinical and pathological characteristics of patient cohort are summarized here. *clear cell RCC only.

Supplementary Table S2

Antibody	Supplier	Clone	Species	Automat	Dilution	Pre-incubation	Incubation time
Apo-Al	Rockland	600-101-109	goat polyclonal	Ventana Discovery	1:1000	32min	44min
ApoB	Abcam	Ab20737	rabbit polyclonal	Ventana Discovery	1:200	32min	60min
SR-BI	Novus	NB400-131	goat polyclonal	Ventana Discovery	1:200	64min	60min
Glut1	Millipore	07-1401	rabbit polyclonal	Ventana Discovery	1:1000	32min	44min
HIF-1α	Abcam	Ab16066	mouse monoclonal	Leica Bond	1:400	60min	30min
CD34	Ventana Roche	790-2927	mouse monoclonal	Ventana Ultra	pre-diluted	16min	32min
CA9	Abcam	Ab15086	rabbit polyclonal	Leica Bond	1:6000	30min	30min
LDLR	Abcam	Ab30532	rabbit polyclonal	Leica Bond	1:6000	30min	30min
VLDLR	Novus	NBP-78162	mouse monoclonal	Leica Bond	1:6000	30min	30min
NRP1	Abcam	EPR3113	rabbit monoclonal	Ventana Ultra Discovery	1:100	90 min	44 min
Cytokeratin	DAKO A/S	AE1/AE3	mouse monoclonal	Ventana Benchmark Ultra	1:50	24 min	32 min
Pax8	Protein Tech Group, Inc.	-	rabbit polyclonal	Ventana Benchmark Ultra	1:400	80 min	60 min

Supplementary Table S2: Immunohistochemistry The table represents the list of the antibodies and the protocol used to perform immunostaining on RCC tissue microarrays.

Supplementary Table S3

Protein of interest	Staining intensity	Tumor stage			
		**pT1/pT2 % (n)**	**pT3/pT4 % (n)**	**Total % (n)**	**p-value**
**apoA-I**	**neg./weak**	35.7% (15)	64.3% (27)	100% (42)	**n.s.**
	**mod./strong**	49.6% (66)	50.4% (67)	100% (133)	
**apoB**	**neg./weak**	42.9% (63)	57.1% (84)	100% (147)	**0.0371**
	**strong**	64.3% (18)	35.7% (10)	100% (28)	
**SR-BI**	**neg./weak**	50.7% (37)	49.3% (36)	100% (73)	**n.s.**
	**mod./strong**	43% (40)	57% (53)	100% (93)	

Supplementary Table S3: Association of apolipoproteins and SR-BI immunoreactivity with pathological tumor stage Percent and absolute (n = number) frequencies of anti-apoA-I, anti-apoB and anti-SR-BI staining intensities among tumor stages.

Supplementary Table S4

Protein of interest	Staining intensity	ISUP grading					
		Grade 1 % (n)	Grade 2 % (n)	Grade 3 % (n)	Grade 4 % (n)	Total % (n)	p-value
apoA-I	neg./weak	0	11.9% (5)	28.6% (12)	59.5% (25)	100% (32)	0.025
	mod./strong	0.8% (1)	25.6% (34)	39.8% (53)	33.8% (45)	100% (133)	
apoB	neg./weak	0.5% (1)	17% (25)	37.4% (55)	44.9% (66)	100% (147)	0.0006
	strong	0	50% (14)	35.7% (10)	14.3% (4)	100% (28)	
SR-BI	neg./weak	1.4% (1)	24.6% (18)	32.9% (24)	41.1% (30)	100% (73)	n.s.
	mod./strong	1.1% (1)	20.4% (19)	41.9% (39)	36.6% (34)	100% (93)	

Supplementary Table S4: Association of apolipoproteins and SR-BI immunoreactivity with tumor grade Percent and absolute (n = number) frequencies of anti-apoA-I, anti-apoB and anti-SR-BI staining intensities among tumor grades.

Supplementary Table S5

Protein of interest	Staining intensity	HIF-1α staining (cell nucleus)			
		Negative % (n)	Positive % (n)	Total % (n)	p-value
apoA-I	neg./weak	70.7% (29)	29.3% (12)	100% (41)	0.0078
	mod./strong	46.9% (61)	53.1% (69)	100% (130)	
apoB	neg./weak	57.3% (82)	42.7% (61)	100% (143)	0.0049
	strong	28.6% (8)	71.4% (20)	100% (28)	
SR-BI	neg./weak	48.6% (34)	51.4% (36)	100% (70)	n.s.
	mod./strong	53.3% (49)	46.7% (43)	100% (92)	

Supplementary Table S5: Association of apolipoproteins and SR-BI immunoreactivity with HIF-1α nuclear staining. Percent and absolute (n = number) frequencies of anti-apoA-I, anti-apoB and anti-SR-BI staining intensities among different categories of nuclear HIF-1α staining.

Supplementary Table S6

Protein of interest	Staining intensity	CA9 staining (cell membrane)			
		Neg./weak % (n)	Strong % (n)	Total % (n)	p-value
apoA-I	neg./weak	28.6% (12)	71.4% (30)	100% (42)	**0.0272**
	mod./strong	13.7% (18)	86.3% (113)	100% (131)	
apoB	neg./weak	18.5% (27)	81.5% (119)	100% (146)	**n.s.**
	strong	11.1% (3)	88.9% (24)	100% (27)	
SR-BI	neg./weak	20.8% (15)	79.2% (57)	100% (72)	**n.s.**
	mod./strong	13% (12)	87% (80)	100% (92)	

Supplementary Table S6: Association of apolipoproteins and SR-BI immunoreactivity with CA9 cell membrane staining Percent and absolute (n = number) frequencies of anti-apoA-I, anti-apoB and anti-SR-BI staining intensities among different categories of CA9 cell membrane staining.

Supplementary Table S7

Protein of interest	Staining intensity	Glut1 staining (cell membrane)			
		**Neg./weak % (n)**	**Strong % (n)**	**Total % (n)**	**p-value**
**apoA-I**	**neg./weak**	52.5% (21)	47.5% (19)	100% (40)	**0.0175**
	**mod./strong**	31.8% (41)	68.2% (88)	100% (129)	
**apoB**	**neg./weak**	39.2% (56)	60.8% (87)	100% (143)	**n.s.**
	**strong**	23.1% (6)	76.9% (20)	100% (26)	
**SR-BI**	**neg./weak**	35.7% (25)	64.3% (45)	100% (70)	**n.s.**
	**mod./strong**	35.3% (30)	64.7% (55)	100% (85)	

Supplementary Table S7: Correlation of apolipoproteins and SR-BI immunoreactivity with GLUT1 cell membrane staining Percent and absolute (n = number) frequencies of anti-apoA-I, anti-apoB and anti-SR-BI staining intensities among different categories of GLUT1 cell membrane staining.

Supplementary Table S8

Protein of interest	Staining intensity	Microvessel density (CD34)				
		Weak % (n)	Moderate % (n)	Strong % (n)	Total % (n)	p-value
**apoA-I**	**neg./weak**	59.5% (25)	38.1% (16)	2.4% (1)	100% (42)	**<0.0001**
	**mod./strong**	27.7% (36)	44.6% (58)	27.7% (36)	100% (133)	
**apoB**	**neg./weak**	37% (54)	45.2% (66)	17.8% (26)	100% (146)	**0.0197**
	**strong**	26.9% (7)	30.8% (8)	42.3% (11)	100% (26)	
**SR-BI**	**neg./weak**	39.4% (28)	42.3% (30)	18.3% (13)	100% (71)	**n.s.**
	**mod./strong**	27.5% (25)	43.9% (40)	28.6% (26)	100% (91)	

Supplementary Table S8: Association of apolipoproteins and SR-BI immunoreactivity with microvessel density (CD34) Percent and absolute (n = number) frequencies of anti-apoA-I, anti-apoB and anti-SR-BI staining intensities among different categories of microvessel density.

Supplementary Table S9

Gene		FLT1 (VEGFR1)	KDR (VEGFR2)	FLT4 (VEGFR3)	NRP1
**HAEC**	Sample 1	3284	27760	374.7	21950
	Sample 2	4239	28660	220.9	22670
	Sample 3	2995	23770	470.1	19550
	**Mean**	**3506**	**26730**	**355.2**	**21390**
**786-O**	Sample 1	0.8	18.9	0.0	4240
	Sample 2	0.7	15.4	0.0	4511
	Sample 3	1.3	9.9	0.7	4355
	**Mean**	**0.9**	**14.7**	**0.2**	**4368.7**
**786-O-VHL**	Sample 1	0.7	12.7	0.7	3004
	Sample 2	0.6	10.7	0.0	3497
	Sample 3	4.2	41.1	0.0	3099
	**Mean**	**1.8**	**21.5**	**0.2**	**3200**

Supplementary Table S9: Expression of VEGF receptors and neuropilin 1 (NRP1) by endothelial cells and the ccRCC cell line 786-O. The expression levels for the VEGF receptors and Neuropilin-1 (NRP1) was assessed by RNA sequencing of human aortic endothelial cells (HAEC = used as positive control), ccRCC parental cell line 786-O and the VHL-transfected cell line 786-O-VHL. The data represents the average numbers of reads in three biological replicates.

Supplementary Figure S1

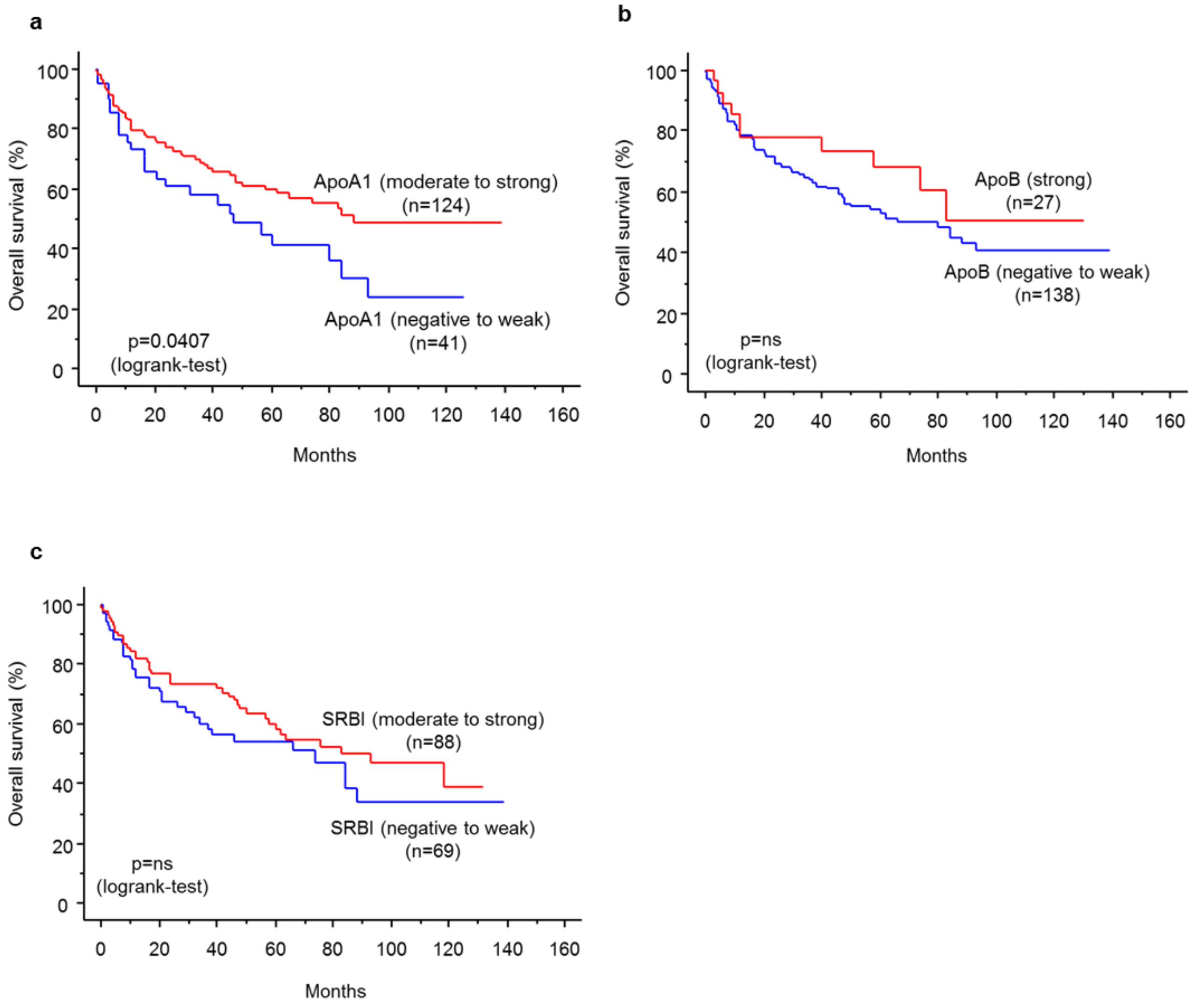

Supplementary Figure S1: Kaplan-Meier curves depicting overall survival (OS) according to differential expression of apolipoproteins and SR-BI in ccRCC tissue microarray sections a, Kaplan-Meier analysis of OS of apoA-I immunoreactivity (*P* value = 0.0407), **b**, Kaplan-Meier analysis of OS of apoB immunoreactivity (*P* value = no significance) and **c,** Kaplan-Meier analysis of OS of SR-BI immunoreactivity (*P* value = no significance).

Supplementary Figure S2

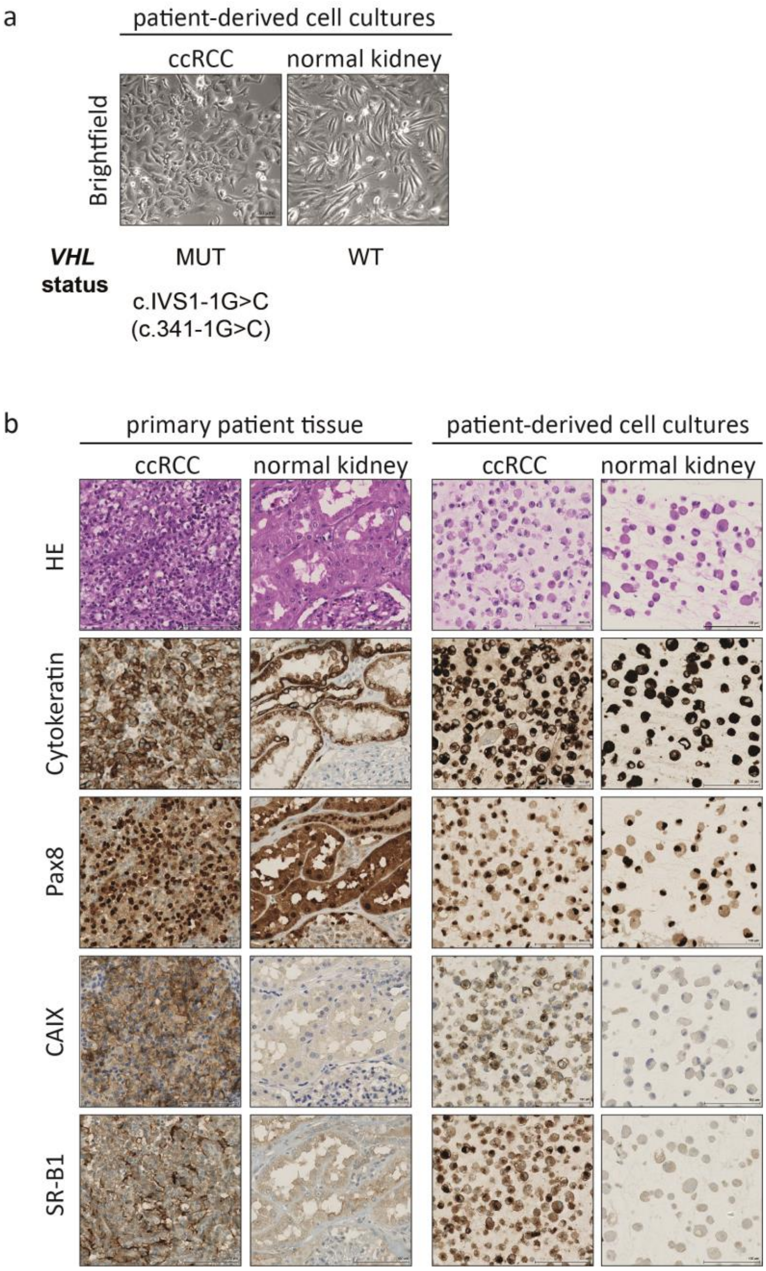

Supplementary Figure S2: Genetic and histological characterization of patient-derived cell cultures. a, Brightfield images (X10 objective) of patient-derived cell cultures depicting the morphology of cells. Scale bar denotes 50 µm. The *VHL* driver mutation was analyzed by targeted sequencing confirming resemblance of cultured ccRCC cells to the primary tumor. **b,** Consecutive sections of FFPE-pellets from patient-derived cell cultures were characterized by HE staining and IHC with the indicated antibodies and compared to the corresponding primary tumors (20X objective).

Supplementary Figure S3

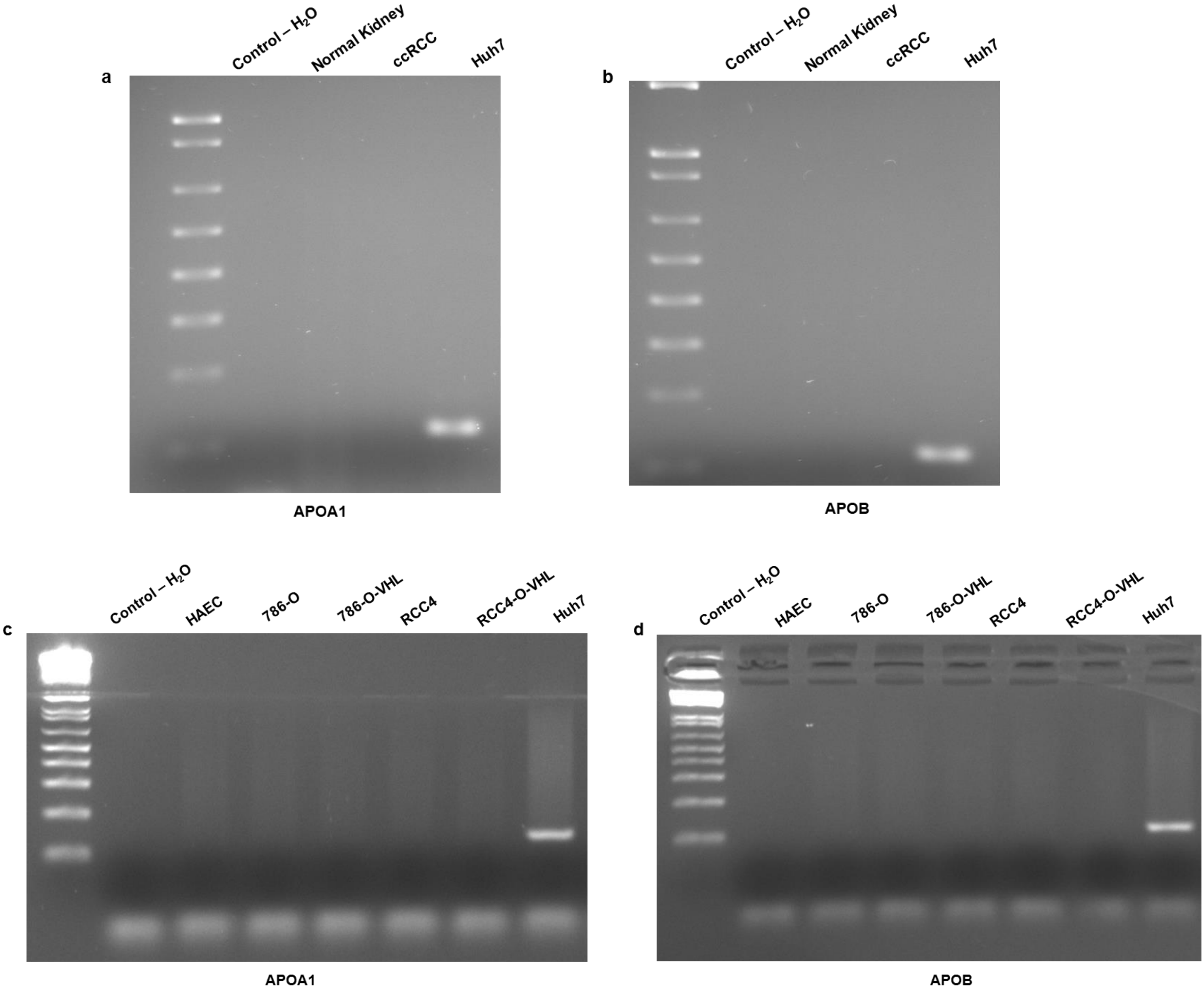

Supplementary Figure S3: mRNA a expression of APO1 and APOB in renal carcinoma cell lines – 786-O and RCC4 cells as well as a patient-derived cell cultures of a clear cell renal cell carcinoma (ccRCC) and normal kidney mRNA levels of APOA1 (113bp, **a,c**) and APOB (111bp, **b,d**) were measured by real-time polymerase chain reaction in 786-O and RCC4 cells compared to Huh7 and HAECs (used as positive and negative controls respectively). Also included were 786-O-VHL transfected cells and RCC4-O-VHL transfected cells. **a, b** Represent the mRNA levels of APOA1 and APOB in the cultured primary cells isolated either from the tumor (ccRCC) or normal kidney compared to Huh7 which was used as positive control. The control reaction was carried out in the presence of water.

Supplementary Figure S4

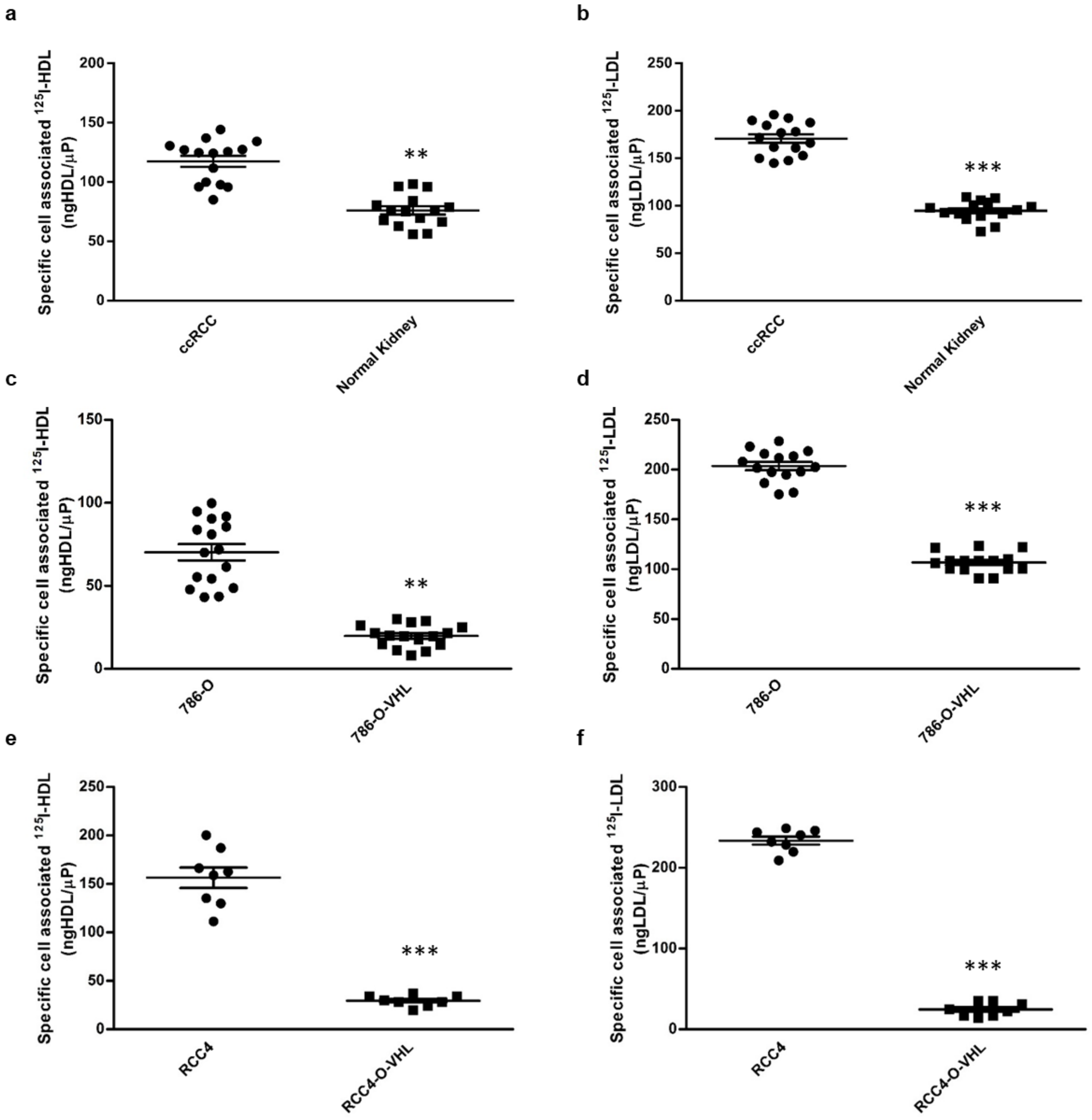

Supplementary Figure S4: Cellular association of radioiodinated lipoproteins with different ccRCC cells (**a, b**) Patient-derived cells from ccRCC or normal kidney (**c. d**), 786-O and 786-O-VHL (**e, f**) RCC4 and RCC4-O-VHL were incubated with 10µg/mL of ^{125}I-HDL (**a, c, e**) or ^{125}I-LDL (**b, d, e**) for 1 hour in the absence (total) or in the presence of 40-fold excess of unlabeled HDL or LDL (unspecific), respectively. Specific association was calculated by subtracting unspecific values from total values. The results are represented as mean±s.e.m of three independent experiments, with two batches of ^{125}I-HDL and ^{125}I-LDL. Significance is determined by Mann-Whitney test. ***$P< 0.001$.

Supplementary Figure S5

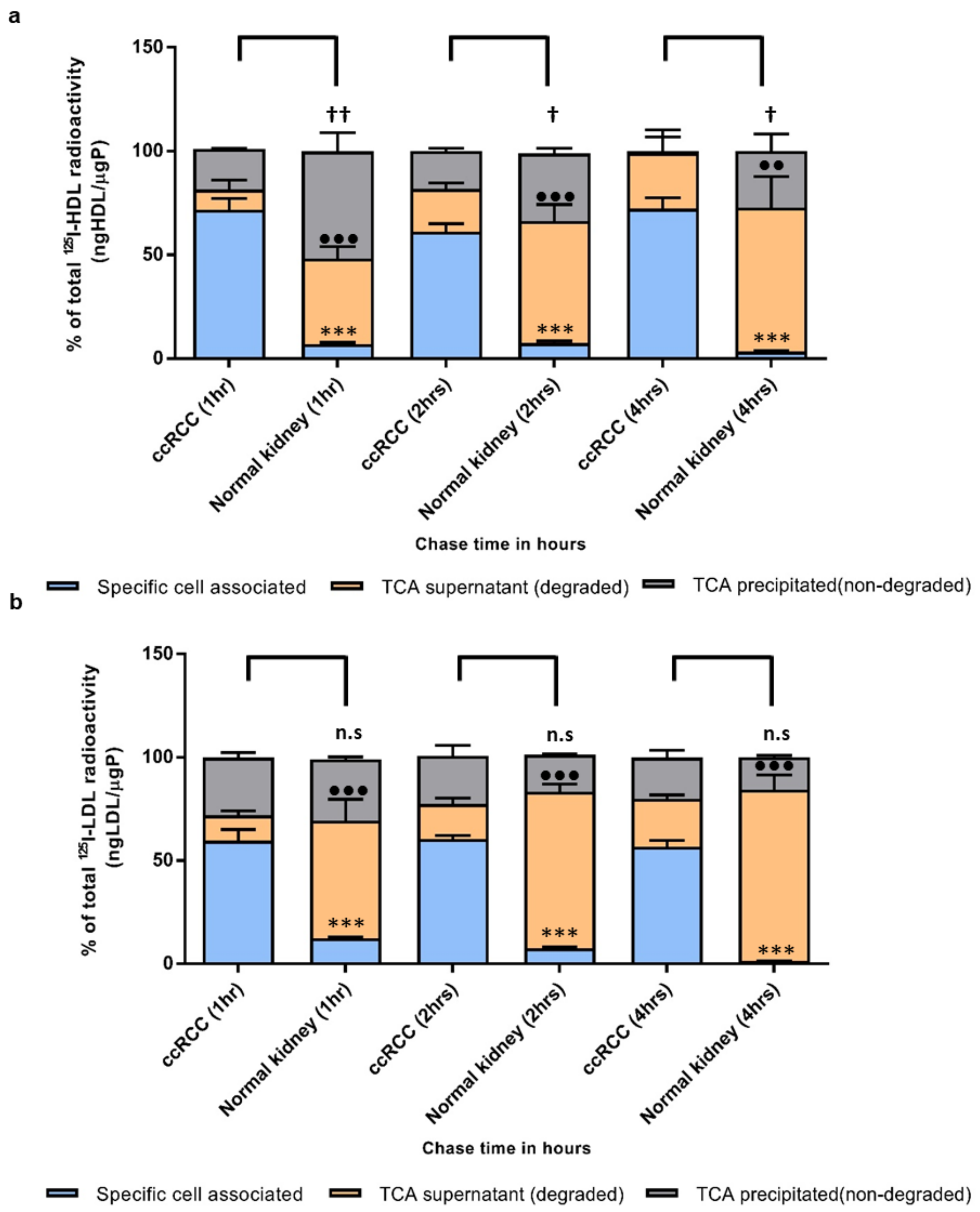

Supplementary Figure S5: Relative Cellular association, resecretion and degradation of (a) ^{125}I-HDL and (b) ^{125}I-LDL in patient-derived ccRCC and normal epithelial kidney cell cultures. Patient-derived ccRCC and normal epithelial kidney cells were pulsed for 1hour with 10µg/mL of either **a,** ^{125}I-HDL or **b,** ^{125}I-LDL at 37ºC in the absence (total) or in the presence of 40-fold excess of unlabeled HDL or LDL (unspecific). Subsequently, the cells were either lysed and analyzed for their content of radiolabel or chased for 1,2 and 4 hours with unlabeled HDL or LDL at 37ºC. The media were collected and subjected to precipitation with TCA to count radioactivity of non-degraded /resecreted lipoproteins in the precipitate and degraded lipoproteins in the supernatant separately. Specific association was calculated by subtracting unspecific values from total values. Fractions and error bars represent percentages of total radioactivity (= radioactivity in cells + TCA supernatant + TCA supernatant = 100%) as means±s.e.m of two independent experiments, with two batches of ^{125}I-HDL and ^{125}I-LDL. Significance is determined for each fraction at each time point using by Mann-Whitney test. For specific cell associated: *** $P\leq 0.001$. For TCA supernatant: •••$P\leq 0.001$, ••$P\leq 0.01$, •$P\leq 0.05$. For TCA precipitated: †† $P\leq 0.01$, †$P\leq 0.05$. n.s represents “not significant”.

Supplementary Figure S6

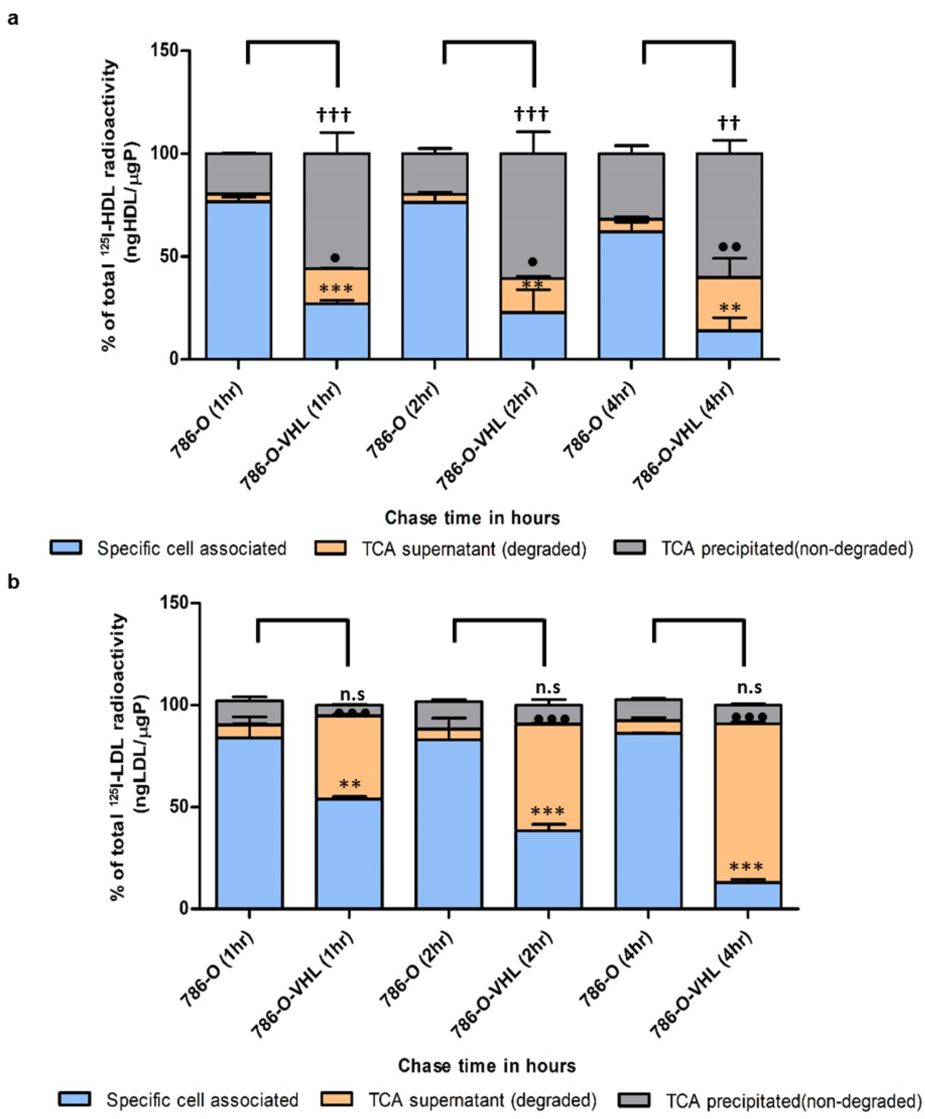

Supplementary Figure S6: Relative cellular association, re-secretion and degradation of (a) ^{125}I-HDL and (b) ^{125}I-LDL in 786-O and 786-O-VHL cells. The results are represented as mean±s.e.m of two independent experiments, with two batches of ^{125}I-HDL and ^{125}I-LDL which were performed as described in the legend of Supplementary Figure S5. Significance is determined for each fraction at each time point using by Mann-Whitney test. For specific cell associated: *** $P\leq 0.001$, ** $P\leq 0.01$. For TCA supernatant: ●●●$P\leq 0.001$, ●●$P\leq 0.01$, ●$P\leq 0.05$. For TCA precipitated: *†††* $P\leq 0.001$, *††*$P\leq 0.01$. n.s represents "not significant".

Supplementary Figure S7

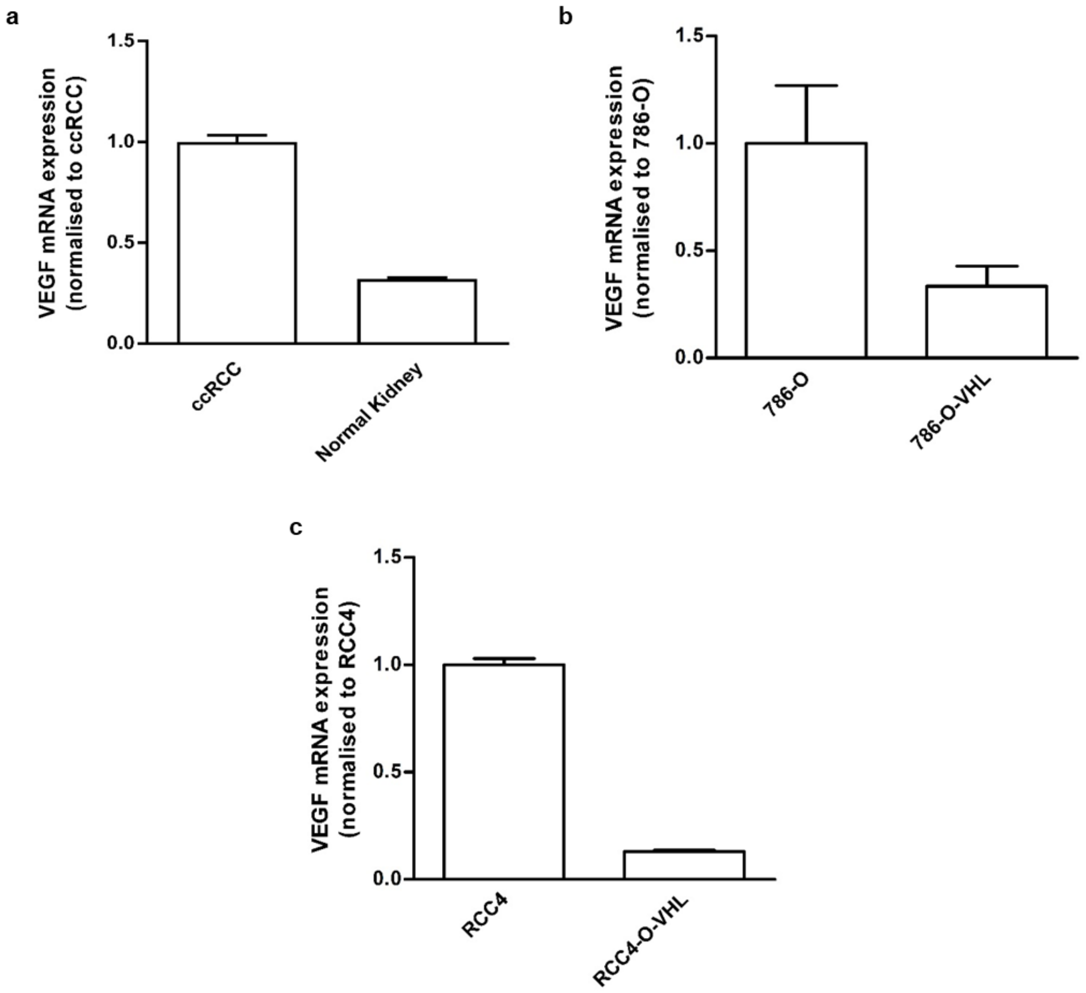

Supplementary Figure S7: mRNA expression VEGF in ccRCC cells Quantification of VEGF mRNA in (**a**) patient-derived ccRCC and normal kidney cell cultures, (**b)** 786-O and 786-O-VHL cells**, (c)** RCC4 and RCC4-O-VHL**.** GAPDH was used a house keeping control gene.

Supplementary Figure S8

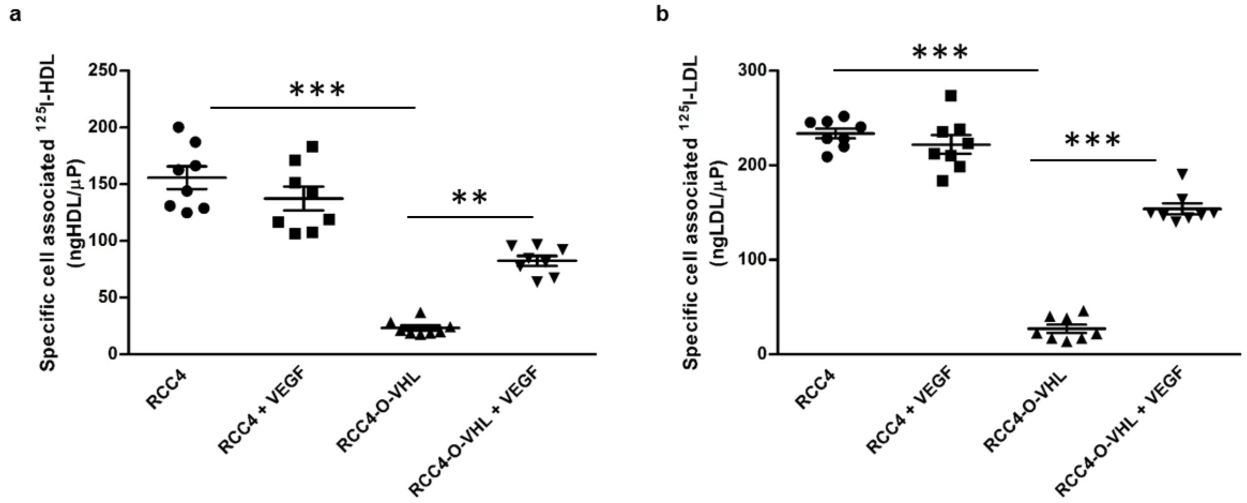

Supplementary Figure S8: Loss of VHL promotes cellular association of ^{125}I-HDL and ^{125}I-LDL in RCC4 cells RCC4 and RCC4-O-VHL cells were pre-treated with 25ng/mL of VEGF for 1 hour prior to assays, followed by incubation with 10µg/mL of (**a**) ^{125}I-HDL or (**b**) ^{125}I-LDL at 37 ºC for 1 hour in the absence (total) or in the presence of 40-fold excess of unlabeled HDL or LDL (unspecific). Specific cellular association was calculated by subtracting unspecific values from total values. The data represent means±s.e.m of two independent experiments with two batches of ^{125}I-HDL and ^{125}I-LDL. Significance is determined by Kruskal-Wallis test followed by Dunn's post-test. ***$P \leq 0.001$, **$P \leq 0.01$, *$P \leq 0.05$.

Supplementary Figure S9

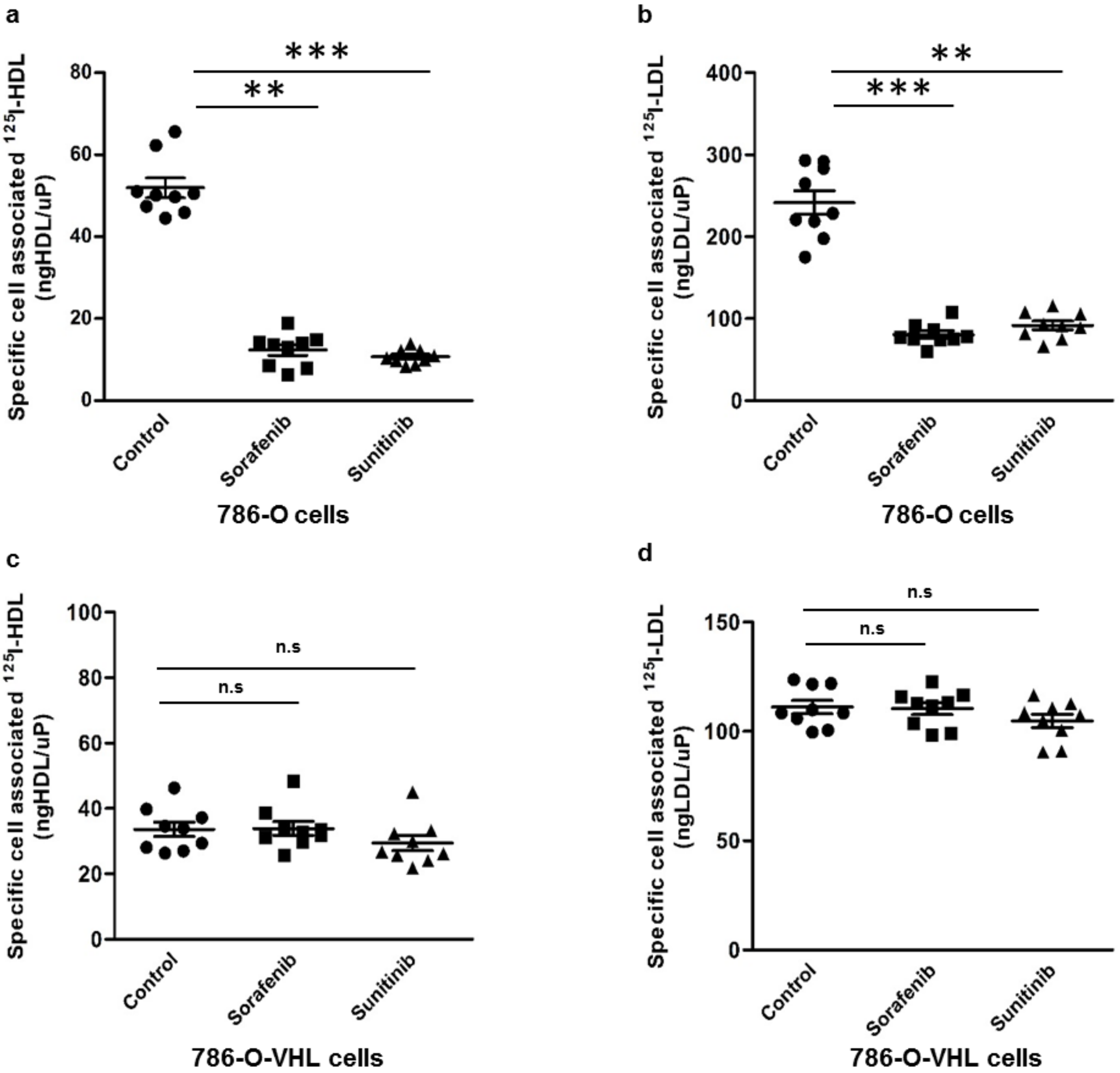

Supplementary Figure S9: Inhibitors of VEGF receptor decrease cellular association of ^{125}I-HDL and ^{125}I-LDL in renal carcinoma 786-O cells (**a, b**) 786-O and (**c, d**) 786-O-VHL cells were pre-treated with either Sorafenib (90nM) or Sunitinib (80nM) for 30 minutes prior to assays, followed by incubation with 10µg/mL of (**a, c**) ^{125}I-HDL or (**b, d**)^{125}I-LDL. at 37 ºC for 1 hour in the absence (total) or in the presence of 40-fold excess of unlabeled HDL or LDL (unspecific). Specific cellular association was calculated by subtracting unspecific values from total values. The data represent means±s.e.m of three independent experiments (each experiment in triplicates), with two batches of ^{125}I-HDL and ^{125}I-LDL. Significance is determined by Kruskal-Wallis test followed by Dunn's post-test. ***$P\leq 0.001$, **$P\leq 0.01$, *$P\leq 0.05$, n.s represents "not significant".

Supplementary Figure S10

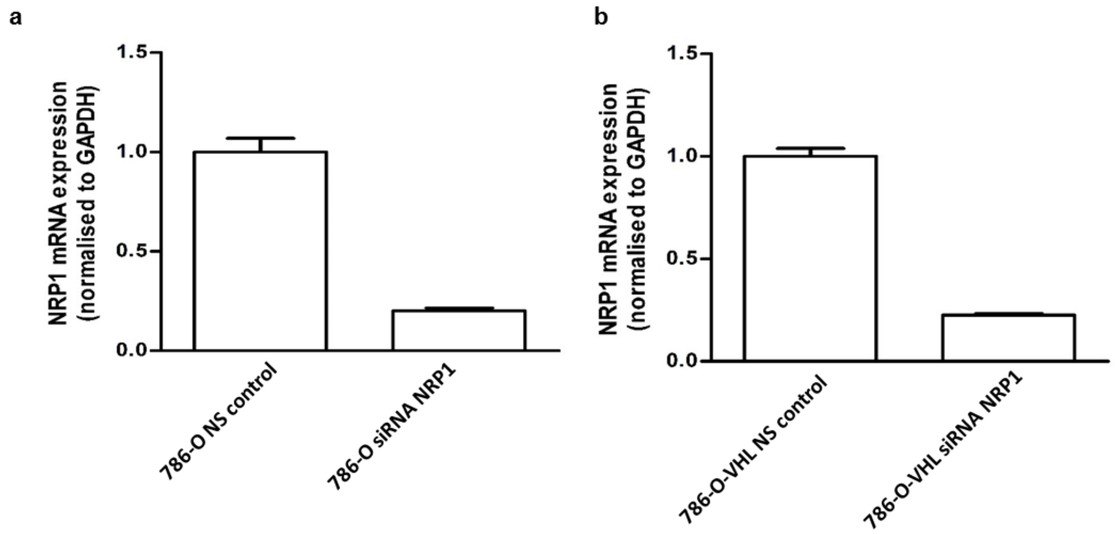

Supplementary Figure S10: Efficiency of NRP1 RNA interference in ccRCC cells (a) 786-O (**b**) 786-VHL cells were transfected with a specific siRNA against NRP1 or with non-silencing control siRNA (NS control) mRNA levels were measured by qRT-PCR after 72 hours of transfection.

Supplementary Figure S11

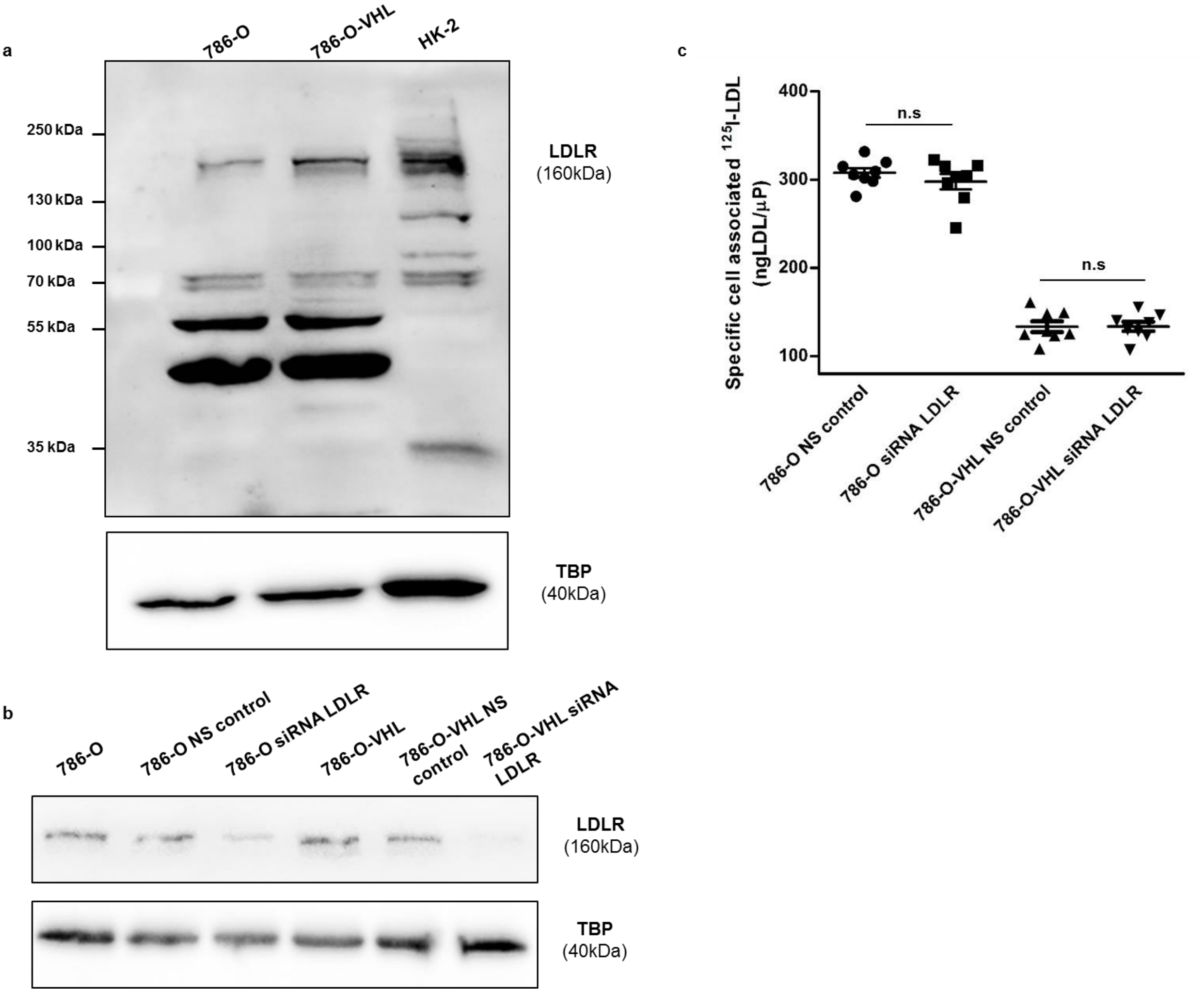

Supplementary Figure S11: Cellular uptake of ^{125}I-LDL in ccRCC is independent of LDLR Western blot analysis of LDLR and TATA-binding protein (TBP) in 786-O and 786-VHL cells compared to healthy kidney cells (HK-2) (**a**). The mature glycosylated LDLR is 160kDa. 786-O and 786-VHL were transfected with siRNA against LDLR or with non-silencing control siRNA (NS control). **(b)** The silencing efficiency was analyzed 72 hours post-transfection at the protein level using western blotting The western blots were probed with anti-LDLR (160kDa) and anti-TBP (40kDa, used as a loading control). **(c)** Specific cell association was analyzed as described in the legend of Figure 2 and in the methods section by incubating ccRCC cells with ^{125}I-LDL at 37 °C for 1 hour. The results represent means±s.e.m of three independent experiments (each experiment in triplicates), with two batches of ^{125}I-LDL. Significance is determined by Kruskal-Wallis test followed by Dunn's post-test. n.s represents "not significant". Complete blots in Supplementary Electrophoretic Blots B,C.

Supplementary Figure S12

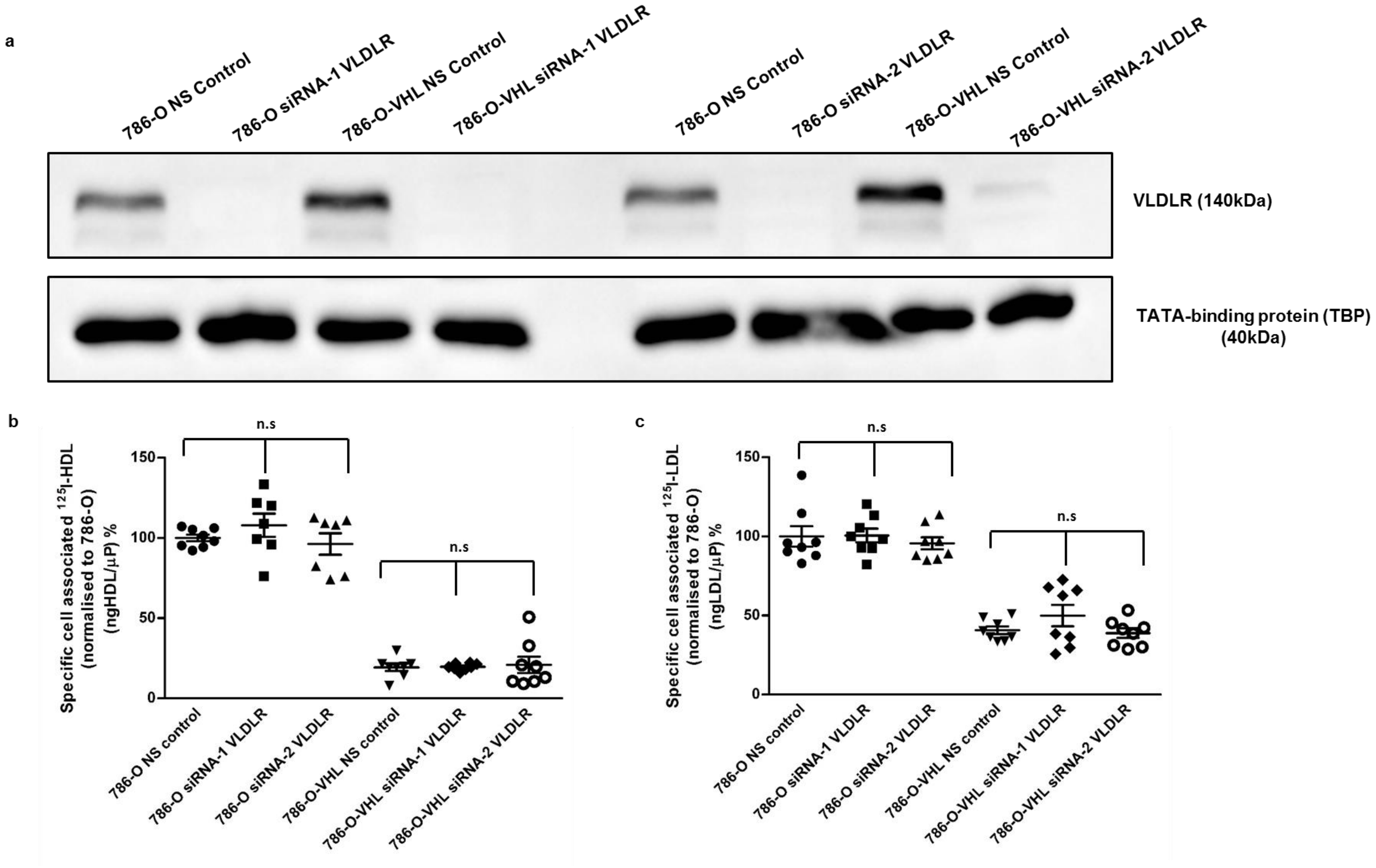

Supplementary Figure S12: Cellular uptake of ^{125}I-HDL and ^{125}I-LDL in ccRCC is independent of VLDLR a, The 786-O and 786-O-VHL cells were transfected with two different siRNA pools targeting VLDLR or with non-silencing control siRNA (NS control). siRNA-1 represents the siGENOME SMARTpool siRNA and siRNA-2 represents the ON-TARGET plus human VLDLR (7436). The silencing efficiency was analyzed 72hours post-transfection at the protein level using western blotting. The western blots were probed with anti-VLDLR (140kDa) and anti-TBP (40kDa, used as a loading control). Specific cell association was analyzed as described in the legend of Figure 2 and in the methods section by incubating ccRCC cells with (**b**) ^{125}I-HDL or (**c**) ^{125}I-LDL at 37 ºC for 1hour. The results represent means±s.e.m of two independent experiments, with two batches of ^{125}I-HDL and ^{125}I-LDL. Significance is determined by Kruskal-Wallis test followed by Dunn's post-test. n.s represents "not significant". Complete blots in Supplementary Electrophoretic Blots D.

Supplementary Figure S13

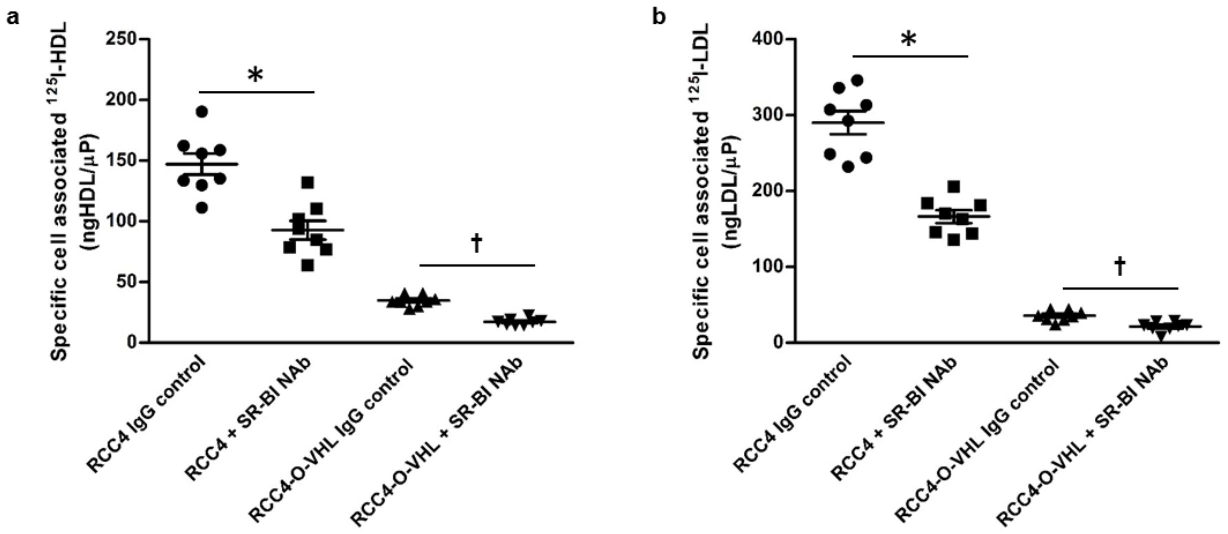

Supplementary Figure S13: SR-BI mediates cellular association of ^{125}I-HDL and ^{125}I-LDL in RCC4 cells. RCC4 and RCC4-O-VHL cells were pre-treated with either anti-SR-BI neutralizing antibody or isotype (IgG) control for 1hour prior to assays, followed by incubation with 10µg/mL of **(a)** ^{125}I-HDL or (**b)** ^{125}I-LDL as described in methods section. The results represent means±s.e.m of two independent experiments with two batches of ^{125}I-HDL and ^{125}I-LDL. Significance is determined by Kruskal-Wallis test followed by Dunn's post-test. IgG control represents isotype control and anti-SR-BI Nab represents treatment with SR-BI neutralizing antibody. *$P \leq 0.05$, †$P \leq 0.05$.

Supplementary Figure S14

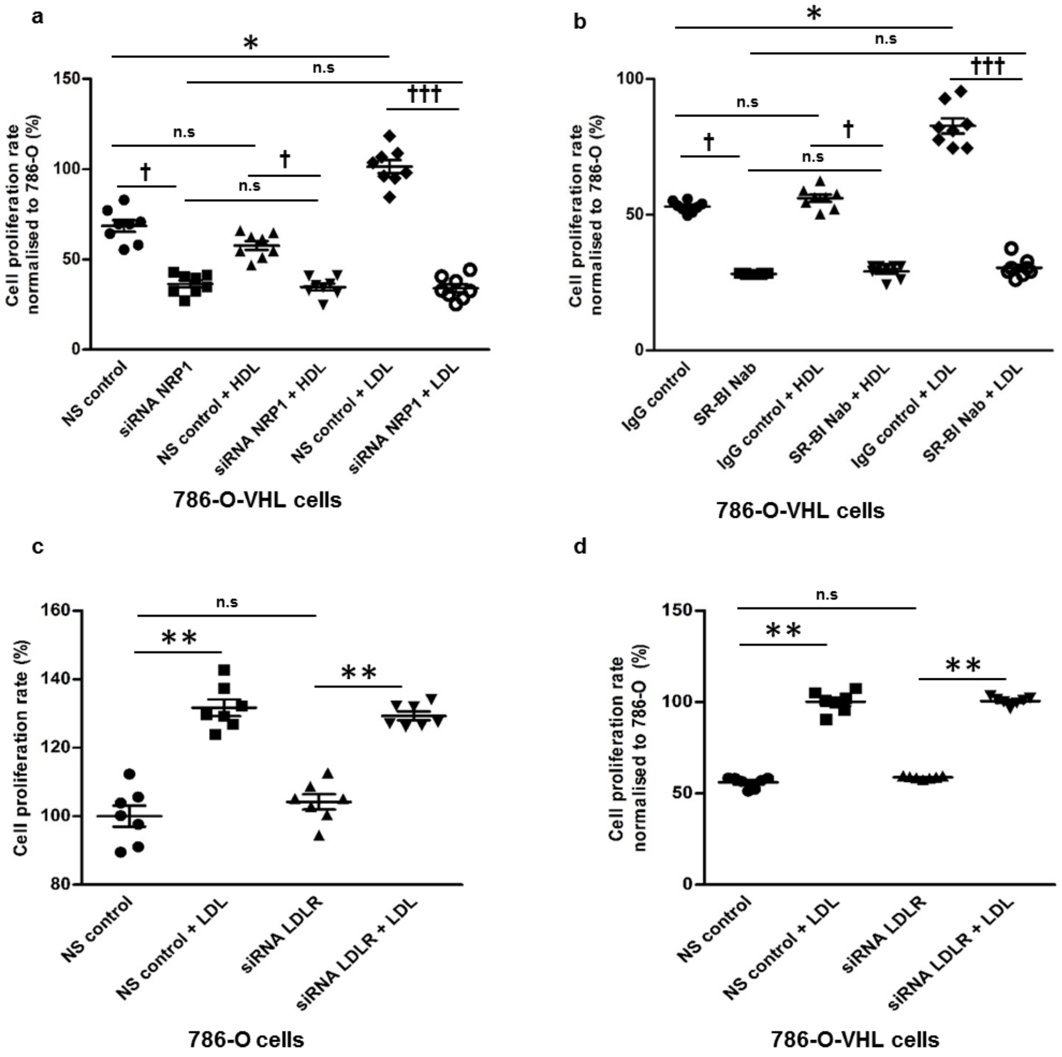

Supplementary Figure S14: Effects of VHL, HDL, LDL, NRP1, SR-BI, and LDLR on the proliferation of ccRCC cells. Proliferation was analyzed using the MTT assay in **(a,b,d)** 786-O-VHL or (**c**) 786-O cells. **(a)** 786-O-VHL cells were transfected with specific siRNA against NRP1 or with non-silencing control siRNA (NS control) for 60hours prior to the overnight treatment with 50µg/mL of HDL or LDL. **(b)** 786-O-VHL cells were pre-treated with neutralizing antibody against SR-BI for 1hour prior to the overnight treatment with 50µg/mL of HDL or LDL. **(c)** 786-O and **(d)** 786-O-VHL cells were transfected with specific siRNA against LDLR or with non-silencing control siRNA (NS control) for 60hours prior to overnight treatment with 50µg/mL of LDL. The results represent means±s.e.m of three independent experiments with two batches of HDL and LDL. Significance is determined by Kruskal-Wallis test followed by Dunn's post-test. IgG control represents isotype control and anti-SR-BI Nab represents treatment with SR-BI neutralizing antibody. ****P*≤ 0.001, ***P*≤ 0.01, **P*≤ 0.05. †††*P*≤ 0.001, ††*P*≤ 0.01, †*P*≤ 0.05 and ns represents "not significant".

Supplementary Electrophoretic Blot A

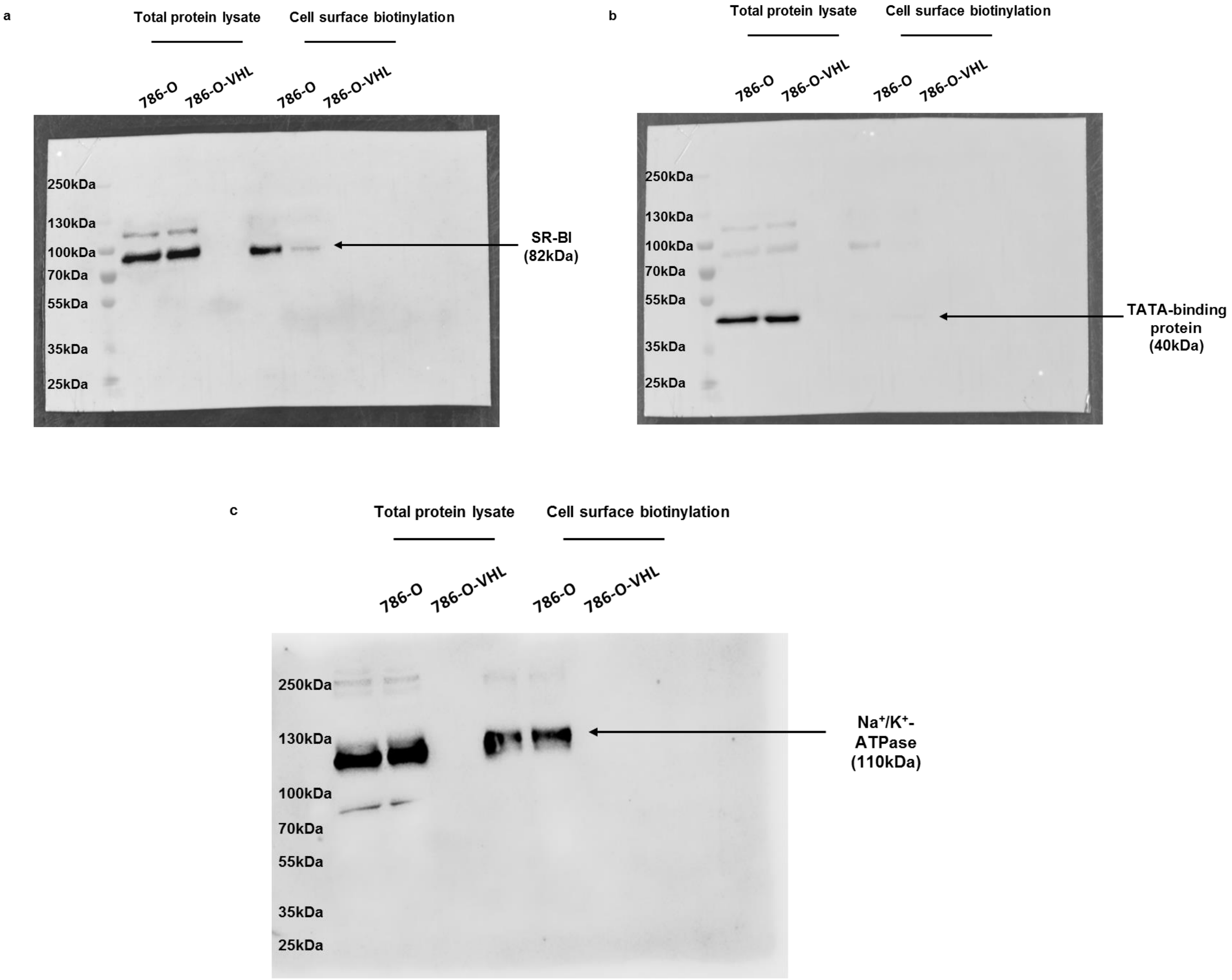

Supplementary Electrophoretic Blot A: Corresponds to the blot shown in Figure 6a

a,Western blot was probed with anti-SR-BI (82kDa). **b,** Western blot was probed with anti-TATA binding protein (TBP, 40kDa, used as intracellular loading control). **c**, Western blot probed with anti-Na^+/K^+-ATPase (110kDa, membrane loading control).

Supplementary Electrophoretic Blot B

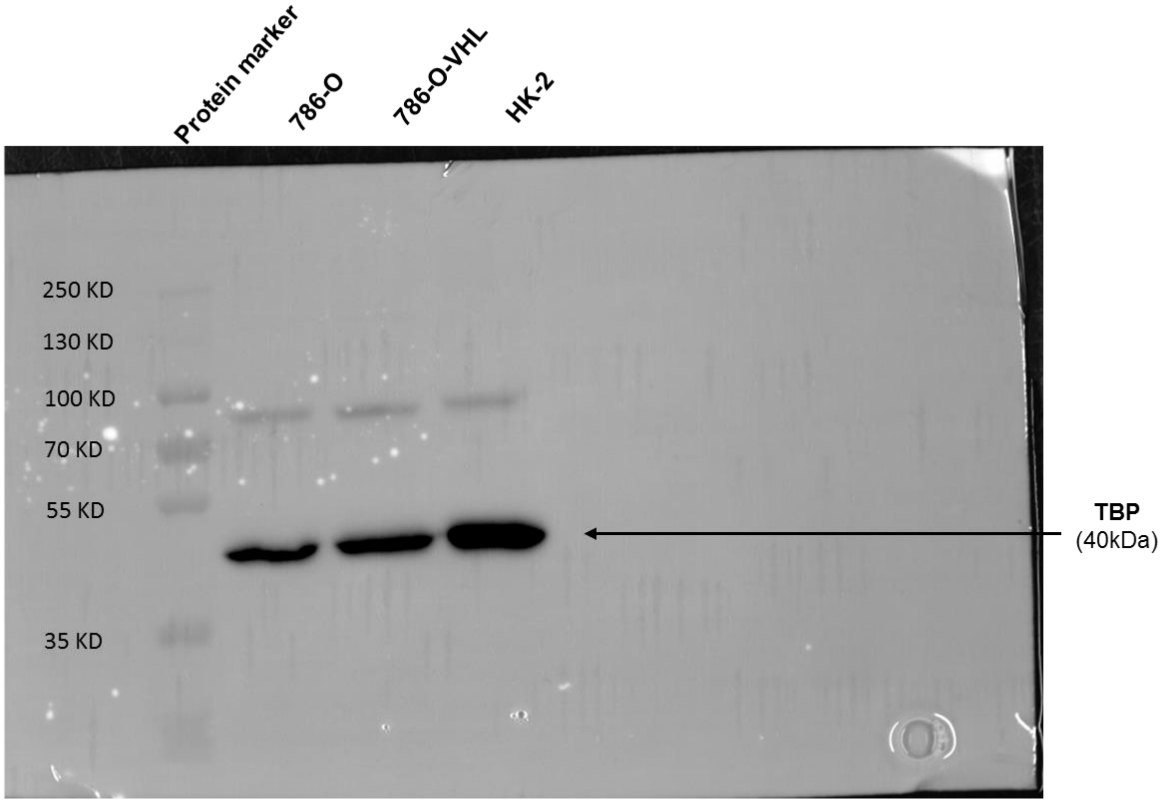

Supplementary Electrophoretic Blot B: Corresponds to blot shown in Supplementary Figure S12a Western blot was probed with anti-TATA binding protein (TBP, 40kDa, used as a loading control).

Supplementary Electrophoretic Blot C

a

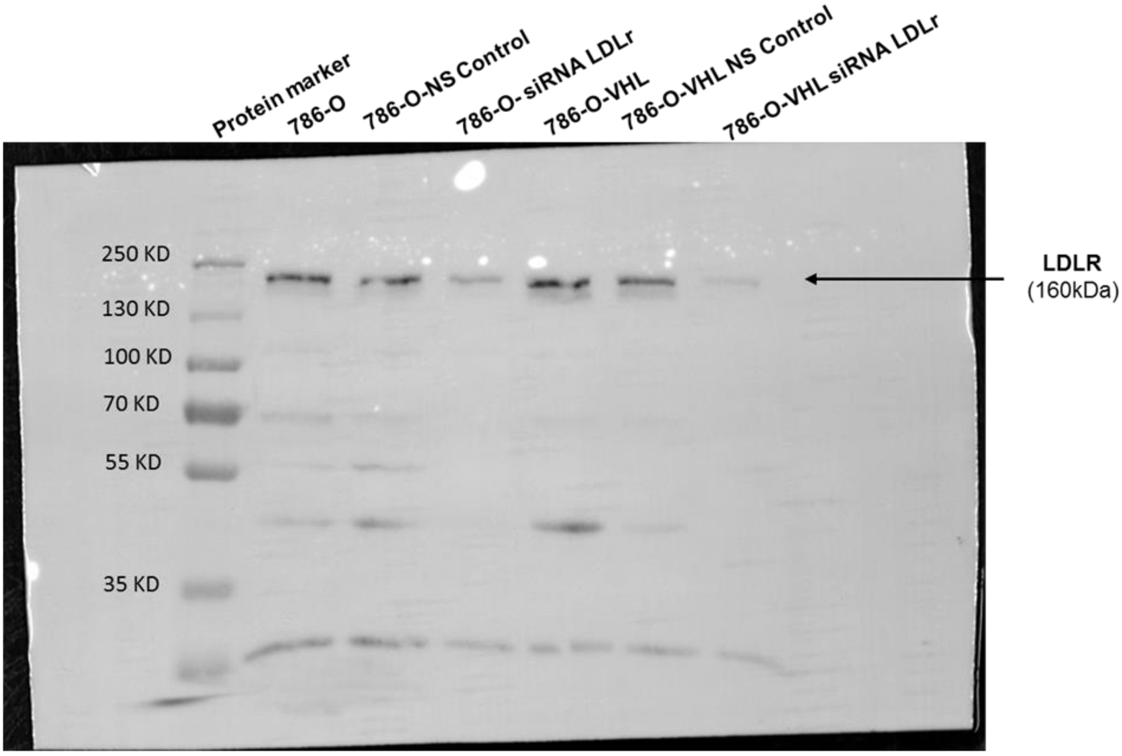

b

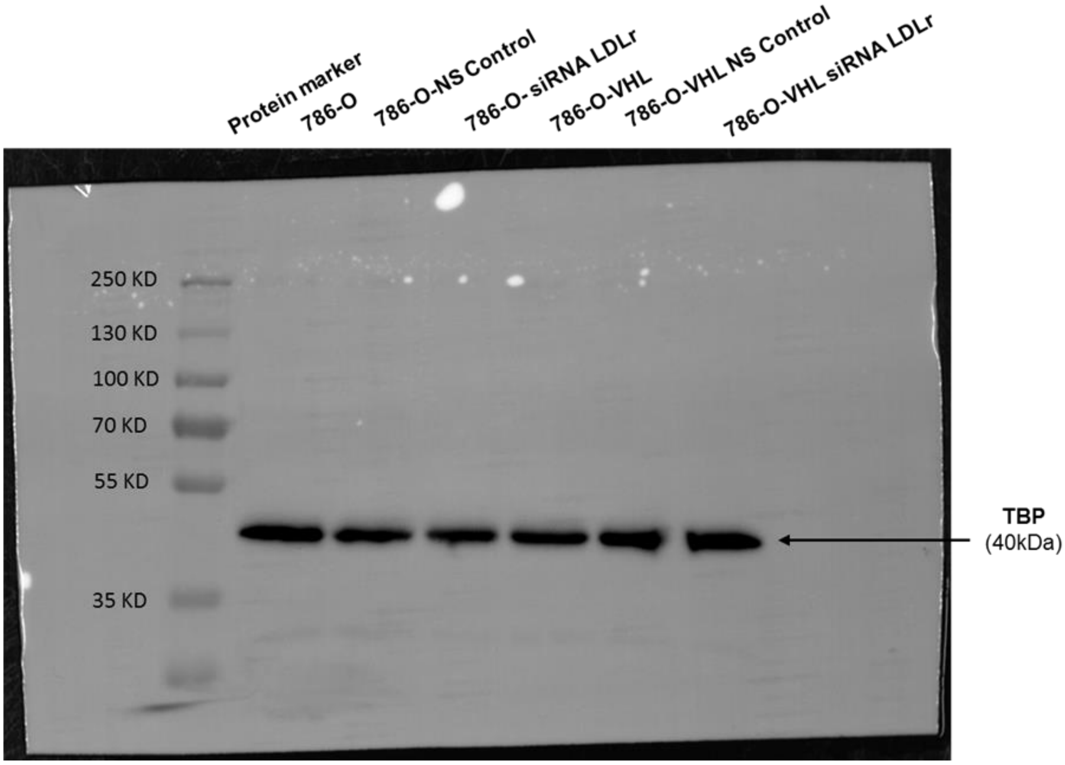

Supplementary Electrophoretic Blot C: Corresponds to the blot shown in Supplementary Figure S12b

a,Western blot was probed with anti-LDLR (160kDa). **b,** Western blot was probed with anti-TATA binding protein (TBP, 40kDa, used as a loading control).

Supplementary Electrophoretic Blot D

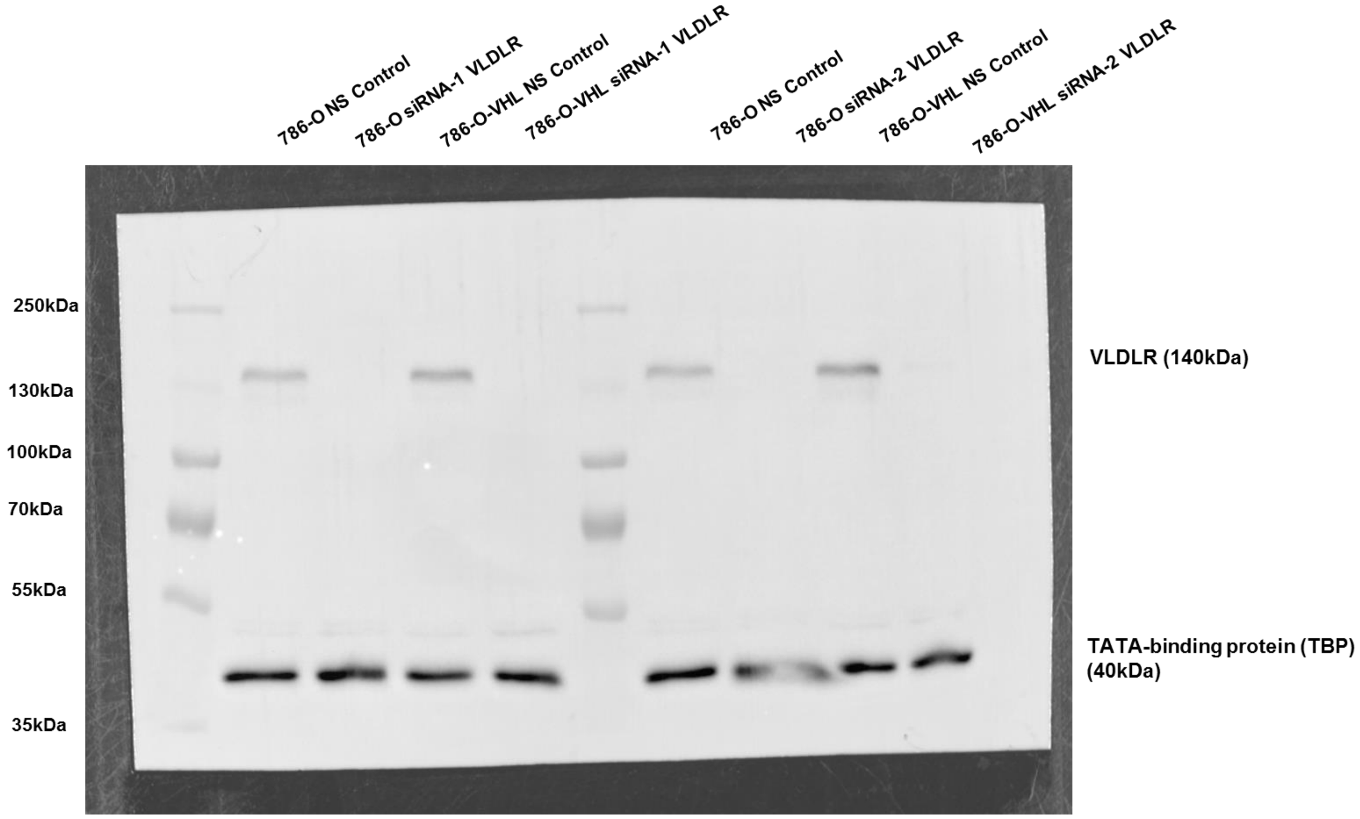

Supplementary Electrophoretic Blot D: Corresponds to the blot shown in Supplementary Figure S13a

Western blot probed with anti-VLDLR (140kDa) and anti-TBP (40kDa, used as a loading control).
